# Preconception care utilization and associated factors among reproductive age women in Mizan-Aman town, Bench Sheko zone, Southwest Ethiopia, 2020. A content analysis

**DOI:** 10.1371/journal.pone.0273297

**Published:** 2022-08-19

**Authors:** Melsew Setegn Alie, Tsedach Alemu, Dereje Alemayehu, Yilkal Negesse, Abebe Gebremariam

**Affiliations:** 1 Department of public health, School of Public Health, College of Health Science, Mizan-Tepi University, Mizan-Aman, Ethiopia; 2 Faculty of Public health, Department of Population and Family health, Institute of health, Jimma University, Jimma, Ethiopia; UNITED STATES

## Abstract

**Background:**

Preconception care is highly important in reducing a number of adverse pregnancy outcomes and helps to improve maternal health. Preconception care optimizes women’s health and improves pregnancy outcomes. It is a cost-effective first-line preventive strategy for birth defects. However, preconception care utilization in Ethiopia was very low. Studies on these issues are limited in Ethiopia in general and in Mizan-Aman town in particular.

**Objective:**

To assess preconception care utilization and associated factors among reproductive age women in Mizan-Aman town, Bench-Sheko Zone, Southwest Ethiopia.

**Methods:**

A community based cross-sectional study design was employed from April 16 to May 26, 2020 in Mizan-Aman town. The total study participants were 624 reproductive age women. Data were collected by using pre-tested interviewer administered questionnaires and entered into Epi-data version 3.1 then exported to STATA version 14 and analyzed accordingly. Univeriate and Bivariable analysis was done by analysis of variance (ANOVA) and independent t-test. Multivariable statistical analysis using generalized linear regression model (GLM) approach was used to classify factors of preconception care utilization. Since our response variable is measured in terms of count variable, we used a Poisson regression model with a log link function. Finally, Statistical significance between dependent and independent variables were assessed by odds ratios and 95% confidence intervals.

**Results:**

Overall, 28.6% of the women receipt atleast one item of preconception care while only 1.5% were taken the whole recommended components of preconception care services. The most common item received in the study area was taking micronutrient supplementation (18.5%). Age of women, educational status, husbands educational status, husbands occupation, wealth status, distance from the health facility, waiting time to get services, planning to pregnancy, age at first pregnancy, previous ANC use, Previous PNC use, adverse pregnancy experience, women’s knowledge of preconception care, and attitude on preconception care were determinants of preconception service utilization.

**Conclusions:**

Preconception care component utilization was lower as compare with recommended service with different disparities. Multipurpose tailored strategies which incorporate a woman with no formal education, poor knwledge on preconception care,never take maternal services previously and distant from health facility could improve preconception care service utilization. Advocative strategies on preconception care component and planning pregnancy may elicite more women to use the services of preconception care.

## Introduction

Preconception care involves on the biomedical, behavioral, and social health interventions to women and couples, which gives the emphasis on provision of preventive, promotive or curative health, and social interventions before the occurrence of conception. It gives to improve the health outcome for the women, newborns, and children. Maternal and child health outcomes are the emphasis area in preconception care [1–3]. To prevent and reverse the adverse maternal and infant health outcome preconception health care promotion has high value and widely acceptable [4]. Know days, all healthcare providers should begin to provide preconception care to every woman every time [5,6]. While in most developing country, the implementation of preconception care is rare and knowledge about its implementation and usage is low but adverse pregnancy outcome is alarmingly increasing [2,3,7]. In some low and middle income countries, such as Bangladesh, Philippines, and Sri Lanka were implementing the guideline for preconception care [2]. Early start of preconception care particularly for girls living in low and middle income countries is very crucial [8]. Many adolescent girls and young women will be thrust into motherhood without the knowledge, skills or support they need while preconception care had solution to such problem [9].

Low or never use of preconception care has several consequences including maternal and neonatal morbidity and mortality, still birth, low birth weight infant, premature delivery, unplanned pregnancy(rapid successive pregnancy) and increase the health care cost [3,10–12]. A study conducted in Ethiopia showed that neural tube defect (27.5%) and hydrocephalus (35.5%) were the major leading cause of admission to the hospital for surgical procedure among children [11]. Preconception care is a critical component of maternal and child health care service and it is a cost-effective first-line preventive strategy for birth defects and other pregnancy-related complications while it is a neglected [2,13,14]. Globally less than 1/3^rd^ of the women of childbearing age visiting the health institution and speak with the health care provider prior to the pregnancy about the health status and its potential impact on pregnancy outcome [2]. In Ethiopia, a number of problems are occurring during pregnancy. Majority of these problems prevented if preconception care is properly implemented with the other continuum of care. For instance anemia during prenatal(31.8%), hepatitis B virus infection among pregnant women(4.7%), poor dietary practices(60.7%), alcohol consumption during pregnancy at least once per weeks(34%), malnutrition among pregnant women(31.8%), mother-to-child transmission of HIV infection(9.93%) [15–20]. Factors identified in previous literatures were age [21–24], educational status [18,25–29], wealth [30],maternal knowledge on preconception car [18,23–25,29–32], planned to pregnancy [18,28,33], positive attitude on preconception care [30] and adverse pregnancy outcome [17,34–36].

In an era of sustainable development, goals (SDG), maternal, newborn and child health still require improvement. To achieve SDG 3 in 2030 care of mothers and newborn is necessary important [16]. Even though preconception care is highly important in reducing a number of adverse pregnancy outcomes and helps to increase other services such as antenatal care and skilled delivery little is known in Ethiopia. Only few studies were done concerning to preconception care in Ethiopia and the existing literature showed that women utilization of preconception care were very low [19,20,37,38]. Even though the concept of preconception care has been explored in maternal and neonatal health as an adjunct to reduce maternal and newborn death in the last 2–3 decades in WHO and CDC [2,3,6] few studies have been examining preconception care utilization in Ethiopia. Investigating the prevalence of preconception care utilization would aid in taking measures for further amendment of service delivery and programs. This in turn allows ministry of health, health sectors and other administrator to design appropriate preconception care policy and implementation strategies. In addition studying on factors of PCC could able to put appropriate and specific solution for each identified factors, which hinder for use of preconception service.

## Materials and methods

### Study design, period and area

A community based cross-sectional study design was conducted in Mizan-Aman town, Bench-Sheko Zone, and Southwest Ethiopia from April 16 to May 26, 2020. The town is located 561 km northeast of Addis Ababa. The town is divided into 5 kebeles that has a total area of 142.71 km with an average elevation of 2840m above sea level. Mizan-Aman town is city administrative with Urban, rural and peri-urban Kebeles. According to the information obtained from the district health office in 2018/19, a total estimated population of 69,453 of which, 32,273 are females. Out of all females, 24,679 were women of reproductive age [15–49 years]. There are one teaching hospital, one health center and five health posts, one university and one college under the government. Mizan-Tepi University teaching hospital is the only teaching hospital in the Bench-Sheko zone that gives charge free service for maternal and neonatal care.

### Study participants

#### Source population

All reproductive age women in Mizan-Aman town were the source population.

#### Study population

All randomly selected reproductive age women in Mizan-Aman town who fulfill the inclusion criteria.

### Inclusion criteria

All reproductive age group women who had a history of pregnancy in last 2 years, lived in Mizan-Aman town for 6 months, and above were included under the study.

### Exclusion criteria

Severely ill, deaf and blind women were excluded in the study.

### Sampling technique and procedure

The sample size was determined by using single population proportion formula with the assumption of 95% confidence level, 4% margin of error and the proportion of preconception care was taken from previous study conducted in West Shoa 38.2% [20].

n = (zα⁄2)^2^*p*(1-p)/d^2^

Where; n = sample size

Zα/2 = 95% confidence interval (1.96)

P = 38.2% taken from the previous study [20]

d = 4% margin of error

Based on the above assumption

n = (1.96)^2^*0.382(1–0.382) (⁄0.04)^2^≈567, considering a 10% non-response rate, so the sample size calculated from single population proportion was 624.

All the five Kebeles of Mizan-Aman were taken. To reach the study unit systematic random sampling technique was used in the Kebeles. The first house was selected randomly and then every16^th^ house for all kebele was asked. The sampling interval of the households in each Kebele was determined by dividing the total number of households in the specific kebele to the allocated sample size. When there was no a reproductive age group woman in the selected house, nearby house was selected and interviewed. In case of more than one eligible woman were encountered in the selected household, a lottery method was used to determine which woman would be interviewed.

### Study variables

Dependent variable: Preconception care utilization.

Independent variables: Socio-demographic characteristics, Obstetric and reproductive health factors, Knowledge towards preconception care, Attitude towards preconception care, Health system related, Preexisting medical condition, women’s autonomy.

### Data collection process

Interviewer-administered structured questionnaires were adapted from different literatures of previous studies [2,4,5,7,10,18–20,30,38,39]. Before distributing the questionnaire to data collector, rechecked for missing was done. Auditing, coding and sorting of the collected questionnaire was done manually every day to check for completeness by principal investigator. The questionnaires clearly understood by the data collector before the data collection. The data were collected by five BSc nurse data collectors and supervised by one BSc public health.

### Survey questionnaires

The questionnaires were prepared based on the previous literatures and CDC preconception care guideline. First, it is prepared in English and translated to local language Amharic with consistent way. The questionnaires retranslated to English by the same translator with consistent approach. The questioners were composed of eight parts, the first part is socio-demographic that comprises of 10 items. Part 2 is obstetric and reproductive health that comprised of 18 items. The third part knowledge of preconception care related, the fourth part is attitude, which contains 11 items of 5-point Likert scale to measure attitude towards preconception care. The response categories are 5-point Likert scales ranging from 1 strongly disagree to 5 strongly agree. During analysis, the Likert scale items were categorized into two response categories to compute women’s attitude on preconception care. All scores were summed and transformed to percentage. The fifth part of questionnaires were related to preconception care utilization, which comprises 18 yes /no format response that measures, the outcome variable. The other parts are preexisting medical illness, health facility related, and wealth of the household. Experts assessed whether the data collection tool measures what it intended to measure, and it was comprehensive enough to collect all the information needed to address the objective of the study **(S1 and S2 Appendix).**

### Data management and analysis

Auditing, coding and sorting of the collected questionnaires were done manually every day to check for completeness. After checking the completeness of the data, the data entered by Epi-data manager version 3.1 and then exported to STATA Version 14 for analysis. Cleaned, coded and checked data were analyzed after checking appropriate assumptions. Descriptive analysis was done for both dependent and independent variables and presented in terms of frequency, mean, percentage and text. Principal component analysis (PCA) was used to compute the wealth index from household asset and utility. Appropriateness of PCA for the items was checked by Kaiser Meyer Olkin (KMO) measure of sampling adequacy. KMO indicated that each variable measure of sampling adequacy was greater than 0.50 and overall KMO was 0.585 and Bartlett test of sphericity showed p<0.001. The case to variable ratio showed that 17.8 to 1 which indicates above the required 5 to 1 ratio. Items with communality <0.50 and have complex structure (≥0.4 loading on more than one component) in the rotated component matrix was removed from analysis. Varimax rotation was employed during factor extraction to minimize cross loading of items on to many factors. Before analysis, the appropriate assumptions were checked for generalized linear regression model. Bivariable analysis was done before preceding to generalize linear regression. Analysis of variance and independent t-test were used as bivariable analysis to select the variables for multivariable generalize liner regression. A variable at p-value of 0.25 and less were eligible ofr multivariable analysis. Multivariable statistical analysis using generalized linear regression model (GLM) with Poisson family and log link function approach was used to classify factors of preconception care utilization. The outcome variable in the current study were count therefore, we consider a Poisson regression model with a log link. The dependent variables variance distribution were equal to mean so the authors used Poisson regression. The mean and variance of the dependent variables were 3.1068 and 3.203 respectively. Charts, graphs and figures displayed the results. Finally, the method of association between dependent and independent variables were done by the odds ratios and 95% confidence intervals. A variable at p value of 0.05 during multivariable generalized linear regression were identified as statistical significant.

### Data quality control

The questionnaires were first prepared in English and then translated to Amharic and re-translated back to English by other person to ensure its consistency and accuracy. Pre-test was carried out in Wacha Maji town on 5% of the sample size, which was 31 reproductive age women for one day. After conducting pre-test, some correction of questionnaires were done on sequences and grammar of it. Internal consistency reliability analysis was carried out and Cronbach’s alpha showed the questionnaire reached acceptable reliability, α = 0.796. Training was given for one day for both data collectors and supervisor. The supervisor supervise the performance of the data collectors on daily basis. The collected data were checked for completeness, consistency and clarity by principal investigator and trained supervisor.

### Ethical statement

Ethical clearance was obtained from Institutional Review Board (IRB) of Jimma University, Institute of health, Faculty of public health. Support letter was obtained from department of population and family health. The ethical review reference number of the study was IRB 00095/2020. The necessary permission was obtained from Mizan-Aman town health office. Anonymity of the participants were kept by not to write their name and individuals information will not be disclosed to other third party and informed verbal consent was obtained from each study participant. For the study participants whose age less than 18 years assent was obtained from their family or husband before preceding next steps. All the study participants informed about the purpose of the study, their right to refuse, and assured about the confidentiality of the information they provide. Verbal consent was obtained by briefly explaining the objective and purpose of the study before the actual data collection. For the guardian or the parents’ verbal assent was also obtained before the actual data collection time. Since this is cross sectional study, which was conducted in rural and urban area written consent is inapplicable due to high illiteracy status of the study participants. The verbal consent obtained and witnessed in both participants accordingly before actual data collection.

### Operation definition

**Preconception care**: The provision of preventive, promotive or curative health and social interventions before conception occurs. Interventions of preconception care could be delivered both in health facilities and in community settings [2].

**Preconception care utilized**: preconception care in this study was consider as count variable by adding all eighteen components of preconception care.

**Components of preconception care**: The components of preconception care in this study was taking folic acid/folate, taking iron or ferrous, screen for hepatitis b, screen for hypertension, screen STI, eat extra meal, preparing diet from different cereal, screen for blood group, discussion with husbands, family planning removal, screen for DM, screen for Anemia, Immunization for tetanus, check husbands health condition, screen for HIV/AIDS before conception, checking weight, consulting others for advice, husband screened for chronic illness.

**Good knowledge on preconception care**: Those who have scored greater than or equal to 50% of correct responses to preconception care knowledge questions [19].

**Pre-existing medical illness**: Women who have medical problems like DM, HTN, HIV/AIDS, asthma, anemia, epilepsy and cardiac problem and follow up at health facility before they become pregnant.

**Adverse pregnancy outcomes**: Pregnancy outcome like low birth weight, preterm birth, congenital anomaly, still birth, abortion and neonatal death [40].

**Attitude towards preconception care:** assessed by 11 items of 5-point Likert scale to measure attitude towards preconception care. The response categories were 5-point Likert scales ranging from 1 strongly disagree to 5 strongly agree. Before analysis negatively worded items were reversely coded. During analysis, the Likert scale items categorized into two response categories to compute women attitude towards preconception care. The total score of the respondents calculated for each of components by adding up the individual item scores and converted to percentage. Those whose scored greater than or equal to 50% were considered as having “positive attitude” towards preconception care, whereas those whose score below 50% were categorized as having “negative attitude” towards preconception care.

**Perceived distance from health facility**: distance from health facility categorized as far and near. Distance from health facility more than two kilometer classified as far distance while less than two kilometer classified as near to health facility.

## Result

### Socio-demographic characteristics

A total of, 605 respondents were included in the analysis for this study with the response rate of 96.95. The mean (±SD) age of the study participants was 34.77(±6.068) years. More than half of the respondents, 324(53.6%) were in the age group of 35–49 years. Majority, 470(77.7%) of the respondents were residing in urban area. More than half 334 (55.2%) of the respondents were Orthodox religion follower. Most 396(65.5%) of the respondents had no formal education. About, 371(61.3%) were housewife by occupation. More than one-fifth,133(22.0%) of the respondents were in the category of fifth quintile **(see Table 1).**

**Table 1:** Socio-demographic characteristics of reproductive age women in Mizan-Aman town, Bench-Sheko Zone, Southwest Ethiopia, 2020(n = 605).

Variables	Category	Frequency (%)
Age of respondents	15–24 years	36(6.0%)
	25–34 years	245(40.5%)
	35–49 years	324(53.6%)
Residence	Urban	470(77.7%)
	Peri-urban	99(16.3%)
	Rural	36(6.0%)
Religion	Orthodox	334(55.2%)
	Protestant	171(28.3%)
	Muslim	77(12.7%)
	Others†	23(3.8%)
Ethnicity	Bench	187(30.9%)
	Amhara	180(29.8%)
	Keffa	91(15.0%)
	Welayita	54(8.9%)
	Oromo	42(6.9%)
	Tigray	36(6.0%)
	others٭	15(2.5%)
Marital status	In marital union	599(99.0%)
	Not in marital union٭٭	6(1.0%)
Educational status	No formal education	396(65.5%)
	Primary(1–8)education	99(16.4%)
	Secondary (9–12)	56(9.3%)
	Tertiary(>12)	54(8.9%)
Main occupation of respondent	Housewife	383(63.3%)
	Employed(Gov’t, private)	167(27.6%)
	Farmer	55(9.1%)
Husband educational status(n = 599)	No formal education	256(42.7%)
	Primary(1–8)education	165(27.5%)
	Secondary(9–12)	113(18.9%)
	Tertiary(>12)	65(10.9%)
Husband occupation (n = 599)	Farmer	255(42.6%)
	Merchant	169(28.2%)
	Employed(Gov’t/private)	95(15.9%)
	Others±	80(13.3%)
Total family size	≤4	272(45.0%)
	>4	333(55.0%)
Wealth index	1^st^ Quintile(Lowest)	123(20.3%)
	2^nd^ Quintile	118(19.5%)
	3^rd^ Quintile	130(21.5%)
	4^th^ Quintile	101(16.7%)
	5^th^ quintile(highest)	133(22.0%)

†Catholic

Juba٭Sheka, Silitsa,٭٭Single, Separated, Divorced, ±Student, Daily worker.

### Obstetric and reproductive health characteristics

Majority, 509(84.1%) of the study participants were multiparous. More than two fifth, 271 (44.8%) of the study participants planned their pregnancy. About, 409(61.6%) of the respondents were used family planning in their lifetime. On the subject of ANC visit more than half, 342(56.5%) of the respondents had attended at least one ANC visit in their nearest pregnancy and of all, 182(53.2%) of the respondents were only attended first and second ANC visit. Regarding to postnatal care use of the respondents, 115(19.0%) of them were used for the nearest delivery and majority, 90(78.3%) of the respondent had attended only one or two PNC visit for their nearest delivery. On the subject of previous adverse pregnancy outcome ever in lifetime, 106(17.5%) of the study participants had history of one or more adverse pregnancy outcome experienced before in their lifetime. Of them, who had adverse birth outcome; 48(45.3%), 31(29.3%), 28(26.4%) and 17(16.3%) of the participants had experience abortion, stillbirth, neonatal death and low birth weight respectively **(See Table 2).**

**Table 2:** Obstetric and reproductive health characteristics of reproductive age women in Mizan-Aman town, Bench-Sheko Zone, Southwest Ethiopia, 2020(n = 605).

Variables	Categories	Frequency (%)
Age at first pregnancy	Less than 18	21(3.5%)
	Greater than or equal to 18	584(96.5%)
Gravidity	Only 1 pregnancy	82(13.6%)
	2–4 pregnancies	394(65.1%)
	≥5 pregnancies	129(21.3%)
Parity	Primipara (one delivery)	96(15.9%)
	Multiparous (≥2 deliveries)	509(84.1%)
Planning to pregnancy	Yes	271(44.8%)
	No	334(55.2%)
Previous use of family planning	Yes	409(61.6%)
	No	196(32.4%)
Types of family planning use(n = 409)	Injectable	222(54.3%)
	Implant	93(22.7%)
	Oral contraceptive	82(20.0%)
	Condom	6(1.5%)
	Post pill	6(1.5%)
Previous ANC visit for nearest pregnancy	Yes	342(56.5%)
	No	263(43.5%)
Number of ANC visit(n = 342)	1–2	182(53.2%)
	3	89(26.0%)
	> = 4	71(20.8%)
Previous PNC visit for nearest delivery	Yes	115(19.0%)
	No	490(81.0%)
Number of PNC visit(n = 115)	1–2	90(78.3%)
	> = 3	25(21.7%)
Previous adverse birth outcome ever	Yes	106(17.5%)
	No	499(82.5%)
Types of adverse pregnancy outcome(n = 106)(multiple response)		
	Ever experience Spontaneous abortion	48(45.3%)
	Ever give still birth	31(29.2%)
	Ever experience neonatal death	28(26.4%)
	Ever give low birth weight	17(16.3%)
	Ever experience congenital anomalies	15(14.2%)
	Ever give preterm baby	12(11.3%)

**NB:** For types of adverse pregnancy outcome, the total summation of percentage is more than 100% due to multiple responses.

### Women’s knowledge on preconception care

**Fig 1:**
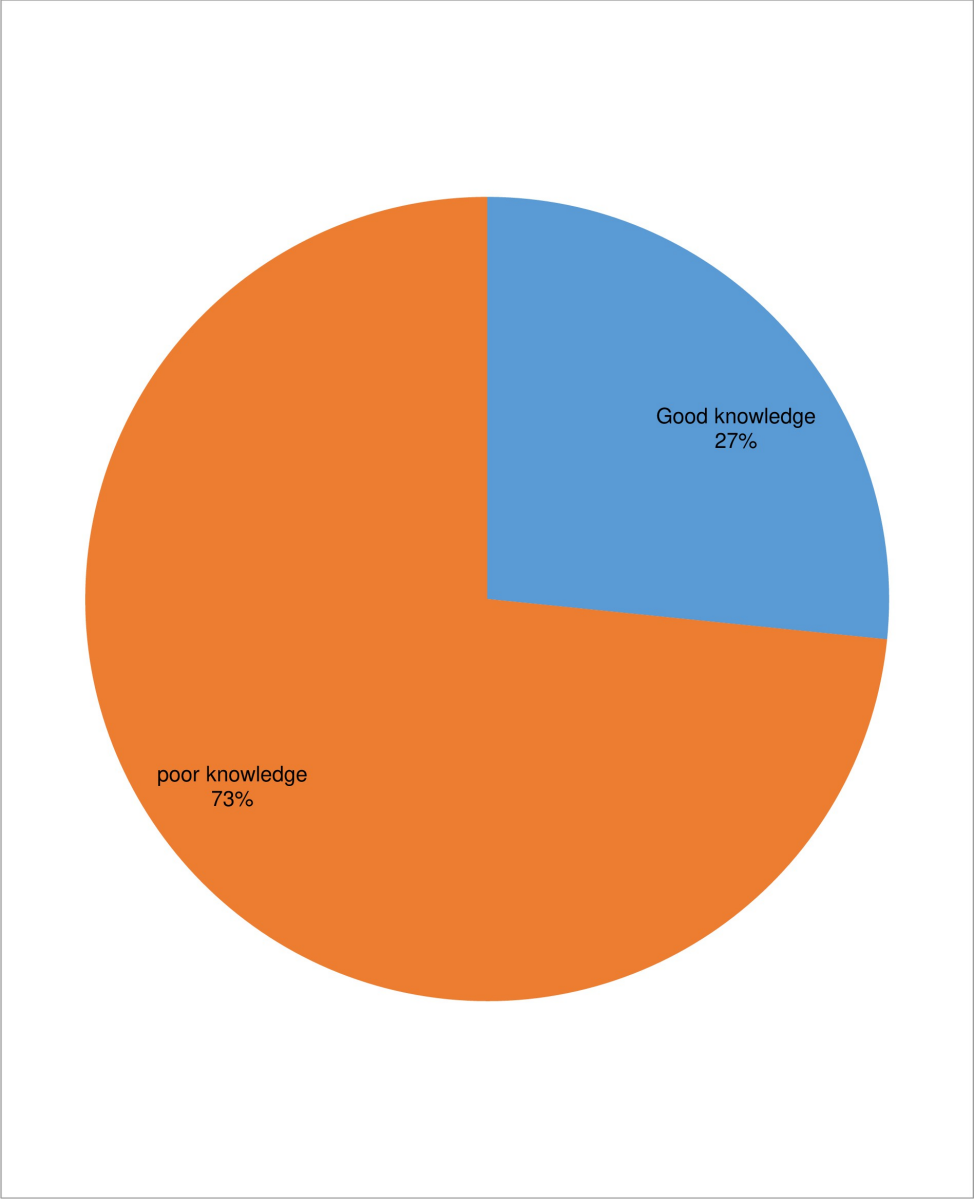
Knowledge of preconception care among reproductive age women in Miza Aman town, Bench-Sheko Zone, Southwest Ethiopia, 2020 (n = 605).

Among the total 605 study participants, 454(75.0%) of the respondents have heard about preconception care. The main source of information was health institution, 186(41.0%), school, 105(23.1%), neighbor, 94(20.7%), and other were heard from mass media, 52(11.5%) and family or friend, 17(3.7%). Overall, the minimum knowledge score of study participant were zero and the maximum score were twenty-four. Of all study participant, one hundred sixty one, 26.6% (95%CI: 23.1, 30.3) of them had good knowledge towards preconception care **(Fig 1).**

### Women’s attitude towards preconception care

**Fig 2:**
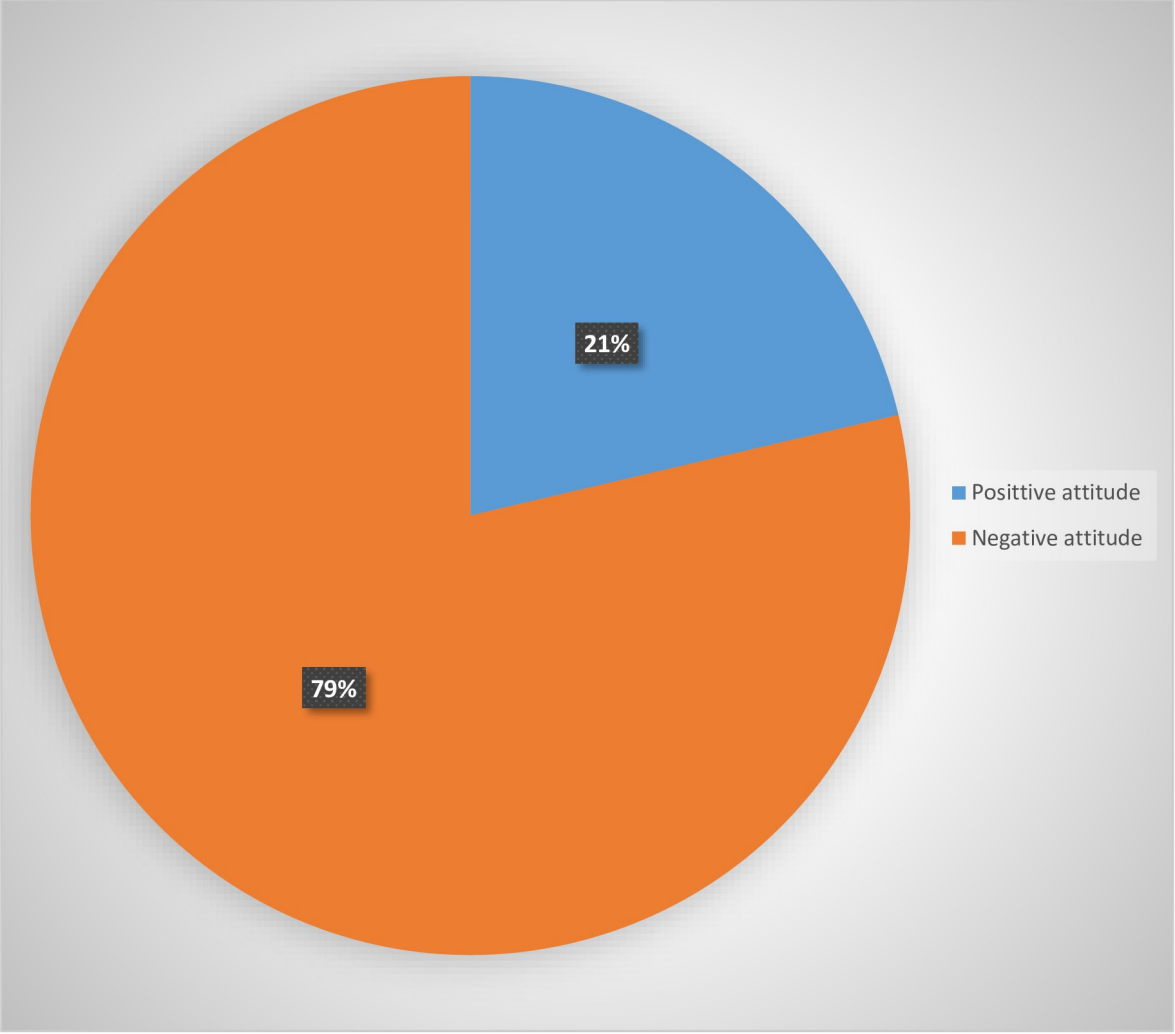
Attitude towards preconception care among reproductive age women in Miza Aman town, Bench-Sheko Zone, Southwest Ethiopia, 2020 (n = 605).

Of the total 605 respondents, 129(21.3.7%) [95% CI: 18.2, 24.7] had positive attitude towards preconception care **(Fig 2).**

### Pre-existing medical illness

**Fig 3:**
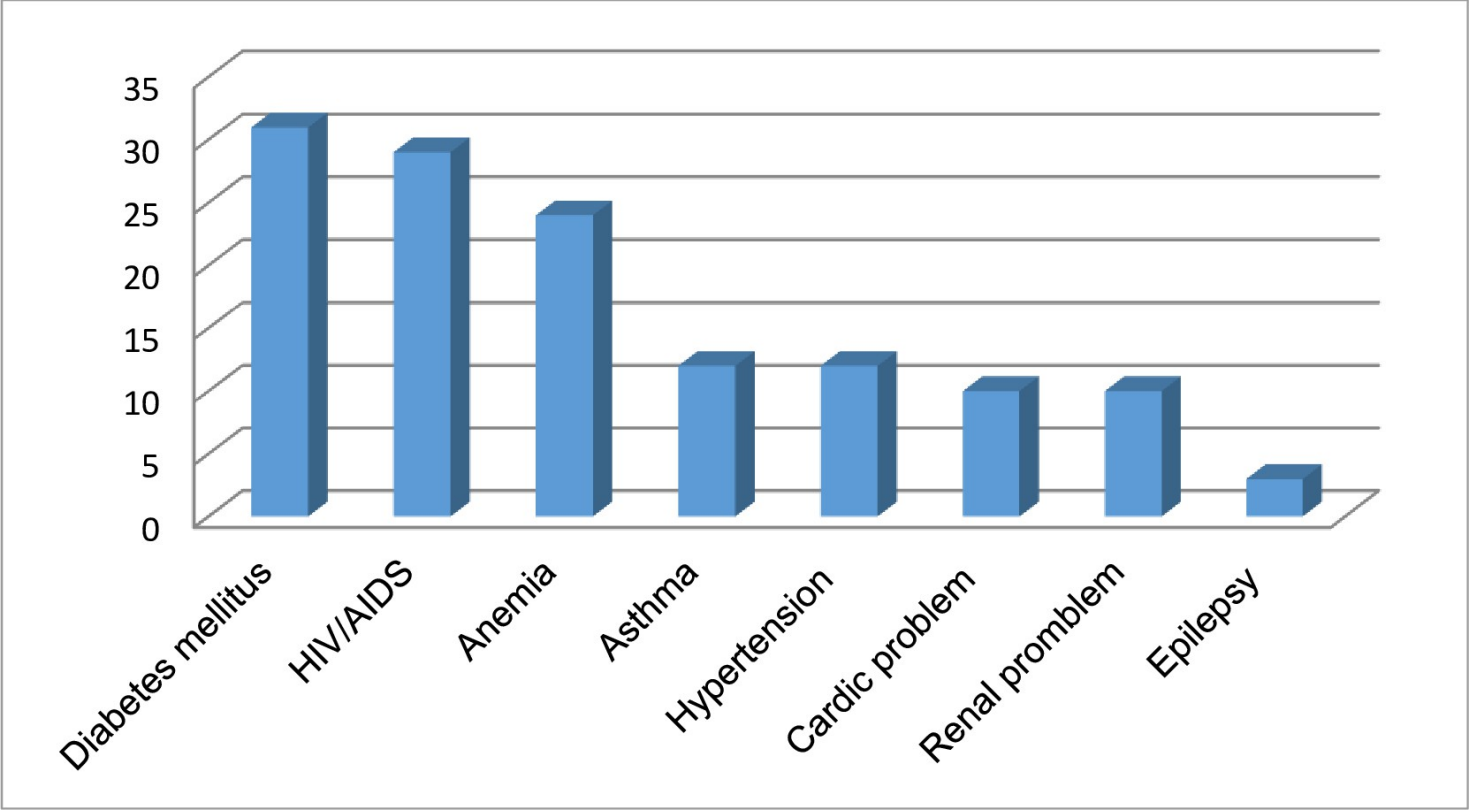
Types of pre-existing chronic illness among reproductive age women in Miza Aman town, Bench-Sheko Zone, Southwest Ethiopia, 2020 (n = 94).

Of the total 605 respondents, 94(15.5%) of them experienced any chronic illness ever in life. The commonest chronic illness were diabetes mellitus, 31(33%), HIV/AIDS, 29(30.9%) while the least common chronic illness is epilepsy, 3(3.2%) **(Fig 3).**

### Health service related characteristics of respondents

About access to the health facility, majority (76.9%) of the respondents were access the health facility with less than two kilometer. The availability of laboratory and adequate medication in nearby health facility assessed in this study indicated that 54.7% and 65.0% respectively. The availability of PCC guidelines was mentioned 60.5% of the study participants. Concerned with availability of preconception care unit, majority (77.5%) of the respondents mentioned the availability of preconception care unit. For decision-making autonomy, the majority of participants (83.6%) were autonomous. Of 605 respondents, 238(39.3%) of the study participants got the service about the sake of healthy pregnancy during their visit in health facility and of them only, 12(5.1%) paid for the services. More than one fifth, 142(23.5%) of the study participants had health insurance **(See Table 3)**.

**Table 3:** Health services related characteristics of respondents in Mizan-Aman town, Southern Ethiopia, 2020(n = 605).

Variables	Category	Frequency (%)
Availability of adequate laboratory service	Yes	331(54.7%)
	No	167(27.6%)
	I do not know	107(17.7%)
Availability of adequate medication	Yes	393(65.0%)
	No	113(18.7%)
	I do not know	99(16.3%)
Availability of PCC unit	Yes	469(77.5%)
	No	136(22.5%)
Autonomy to maternal health service	By joint decision	238(39.3%)
	By self-decision	268(44.3%)
	By husband decision	99(16.4%)
From whom you receive health care access assistance	From my husband	252(41.7%)
	From relatives	183(30.2%)
	From families	116(19.2%)
	From neighbors	54(8.9%)
Time to reach health facility(by public transport)	≤30 minutes	406(67.1%)
	>30 minutes	199(32.9%)
Distance from the health facility(How much far away)	≥2km	140(23.1%)
	<2km	465(76.9%)
Availability of Guideline	Yes	366(60.5%)
	No	146(24.1%)
	I do not know	93(15.4%)
Getting service for sake of healthy pregnancy	Yes	238(39.3%)
	No	367(60.7%)
Payment for services of healthy pregnancy(n = 238)	Yes	12(5.1%)
	No	226(94.9%)
Affordability of payment(n = 12)	Fair	2(16.7%)
	Cheap	10(83.3%)
Using health insurances	Yes	142(23.5%)
	No	463(76.5%)
Health care provider tell care before pregnancy	Yes	291(48.1%)
	No	314(51.9%)

### Preconception care component utilization

Concerning to the component of preconception care, women utilized some components more relative to the others, the most repeatedly used component were taking of iron or ferrous, 80 (13.2%), removing family planning, 108(17.9%), and screening for sexually transmitted infection, 58(9.6%) while the least reported component that used by study participants were consulting any one for advice, 27(4.5%) **(See Table 4).**

**Table 4:** Preconception care component utilization among reproductive age women in Mizan-Aman town southwest Ethiopia, 2020(n = 605).

Variable	Categories	Frequency (%)
Taking folic acid/folate	Yes	32(5.3%)
	No	573(94.7%)
Family planning removal	Yes	108(17.9%)
	No	497(82.1%)
Taking iron or ferrous	Yes	80(13.2%)
	No	525(86.8%)
Screen STI	Yes	58(9.6%)
	No	547(90.4%)
Eat extra meal	Yes	58(9.6%)
	No	547(90.4%)
Screen for DM	Yes	46(7.6%)
	No	558(92.4%)
Screen for Anemia	Yes	51(8.4%)
	No	554(91.6%)
Screen for hepatitis b	Yes	50(8.3%)
	No	555(91.7%)
Immunization for tetanus	Yes	49(8.1%)
	No	556(91.9%)
Screen for blood group	Yes	49(8.1%)
	No	556(91.9%)
Screen for hypertension	Yes	46(7.6%)
	No	559(92.4%)
Preparing diet from different cereal	Yes	45(7.4%)
	No	560(92.6%)
Check husbands health condition	Yes	39(6.4%)
	No	566(93.6%)
Screen for HIV/AIDS before conception	Yes	38(6.3%)
	No	567(93.7%)
Checking weight	Yes	37(6.1%)
	No	568(93.9%)
Consulting others for advice	Yes	27(4.5%)
	No	578(95.5%)
Discussion with husbands	Yes	41(6.4%)
	No	561(93.6%)
Husband screened for chronic illness	Yes	39(6.8%)
	No	566(93.2%)

### Factors associated with preconception care utilization

Based on the descriptive analyses of one-way anova (ANOVA) and independent t-test sixteen variables selected for generalized linear model. The variable with the p-value less than 0.25 were selected for the final model. Results of the multivariable generalized linear regression analysis with a family of Poisson and log link function identified; fourteen variables defined as factors of the preconception care service use. The level of education attained by the mother was found to have a significant positive relationship with the receipt of preconception care services. Mothers with secondary education were 41% higher chance [AOR 1.41, 95% CI 1.16, 1.74] of utilizing PCC component items as compare with mothers with no formal education. Women attained tertiary education were 2.16 times [AOR 2.16, 95% CI 1.8, 2.6] more likely to receive preconception care component as compared with women without formal education. Maternal age was one of significant factors of preconception care service use. Younger age women were more likely utilized preconception care than older aged women. Mothers age of 15 to 24 years old 48% more likely receipt preconception care services with mothers age of 25 to 34 years old [AOR 0.52, 95% CI 0.38, 0.70]. Husbands’ education and occupation were the determinants of preconception care services. Husbands attained primary and secondary education were 33% and 20% less likely received PCC services of his wife respectively. The household wealth were one of the determinants of preconception care services use. The wealth of the households were positively associated with receipt of preconception care services. The second, third, fourth and fifth quantile wealth were 2.5, 1.4, 1.6 and 1.5 times more likely received PCC service items [AOR 2.35, 95%CI:1.87,2.97], [AOR 1.39, 95%CI 1.09,1.77], [AOR 1.57, 95%CI 1.20,2.06] and [AOR 1.5, 95%CI 1.16,1.93] as compare with the lowest(first) quantile households respectively. Perceived time spent to receive the services in health facilities found as a determinant of preconception care service use. Mothers who spent less than 30 min to get a service were 1.4 times more likely to use PCC [AOR 1.4, 95% CI 1.19,1.66] as compare with counterpart.

Women age at first pregnancy were other determinants to receive preconception care services. Women’s age greater than 18 years at first pregnancy were 2.6 times more [AOR 2.58, 95%CI 1.34, 4.96] received preconception care as compare with women who experienced their first pregnancy below 18 years old. Previous maternal health service use were positively associated with preconception care service usage. Previous use of ANC and PNC were significantly associated with preconception care use. Mother who never attended ANC and PNC were 17% and 33% less likely attained preconception care services respectively. Mother who never had a plan to pregnancy were 55% less likely to receive preconception care as compared with who had plan to pregnancy. Previous experience of adverse pregnancy outcome were associated with preconception care use. Mother never experienced adverse pregnancy outcome were 68% [AOR 0.32, 95% CI 0.27, 0.36] less likely use PCC services. Furthermore, maternal knowledge of PCC was also an important predictor of the receipt of PCC items. Respondents with poor knowledge of PCC have had a 47% less chance of utilizing recommended items of PCC service [AOR 0.53, 95% CI 0.46, 0.61] as compare with counterpart. Attitude of the women on preconception care were one of factors of PCC service use. Women with positive attitude were 40% higher chance of utilizing preconception care services [AOR 1.4, 95%CI 1.17,1.69] as compare with women with negative attitude (**See Table 5**).

**Table 5:** Factors of preconception care utilization among reproductive age women using multivariable generalized linear regression model with Poisson link in Bench Sheko Zone, southwest Ethiopia, 2020.

Variable categories		Mean of a component preconception care	p-value^c^	AOR(95%CI)	P-value*
Age of respondents	15–24 years	1.61	0.005^a^	1	1
	25–34 years	0.93		0.52[0.38,0.70]	**0.000**
	35–49 years	1.90		0.91[0.67,1.2]	0.56
Educational status	No formal education	1.08	0.000^a^	1	1
	Primary education	1.17		1.05[0.84,1.3]	0.67
	Secondary education	2.86		1.41[1.16,1.74]	**0.001**
	Tertiary education	3.65		2.16[1.8,2.6]	**0.000**
Husbands educational status	No formal education	1.42	0.209^a^	1	1
	Primary education	1.18		0.67 [0.56,0.81]	**0.000**
	Secondary education	1.60		0.80[0.66,0.98]	**0.034**
	Tertiary education	2.24		0.91[0.74,1.13]	0.422
Husbands occupation	Farmer	1.43	0.022^a^	1	1
	Merchant	2.08		1.23[1.05,1.4]	**0.009**
	Employed(Gov’t/private)	1.18		0.99[0.78,1.25]	0.96
	Others±	0.70		0.84[0.63,1.13]	0.26
Wealth index	1^st^ Quintile(Lowest)	0.90	0.001^a^	1	1
	2^nd^ Quintile	2.66		2.35[1.87,2.97]	**0.000**
	3^rd^ Quintile	1.50		1.39[1.09,1.77]	**0.008**
	4^th^ Quintile	1.22		1.57[1.20,2.06]	**0.001**
	5^th^ quintile(highest)	1.17		1.5[1.16,1.93]	**0.002**
Distance from health facility	< = 2KM	1.05	0.000^b^	1	**1**
	>2KM	2.93		0.56[0.48,0.65]	**0.000**
Preconception care unite availability	Yes	1.44	0.204^b^	0.97[0.81,1.15]	0.713
	No	1.63		1	1
Waiting time to services	< = 30minute	1.64	0.000^b^	1.4 [1.19,1.66]	**0.000**
	>30 minute	1.18		1	1
Age at first pregnancy	< = 18 years	0.48	0.02^b^	1	1
	>18 years	1.52		2.58[1.34,4.96]	**0.004**
Planning to pregnancy	Yes	2.46	0.000^b^	1	1
	No	0.69		0.45[0.38, 0.53]	**0.000**
Previous ANC use	Yes	1.94	0.000^b^	1	1
	No	0.89		0.83[0.70,0.97]	**0.028**
Previous PNC use	Yes	2.26	0.000^b^	1	1
	No	1.30		0.67[0.57,0.79]	**0.000**
Previous adverse outcome	Yes	4.26	0.000^b^	1	1
	No	0.90		0.32[0.27,0.36]	**0.000**
Women’s knowledge on PCC	Good	2.83	0.000^b^	1	1
	Poor	1.00		0.53[0.46,0.61]	**0.000**
Women’s attitude on PCC	Positive	1.12	0.008^b^	1.40[1.17,1.69]	**0.000**
	Negative	1.59		1	1

Key 1: Reference.

±Student, Daily worker.

AOR: Adjusted odds ratio.

p-value^c^ indicates p-value at descriptive analysis.

P-value*indicates p-value at generalized linear model with Poisson log link.

P-value with^a^ indicate descriptive analysis by using ONE WAY ANOVA.

P-value with^b^ indicates independent t-test analysis.

## Discussion

The current study found that only 9(1.5%) of respondents received all the eighteen WHO and CDC recommended items of preconception care services before they underwent their last pregnancy. The mean (± SD) score for receiving preconception care service items before their last pregnancy was 5.2(± 4.93). This finding implies that most of study participants did not implement the elements of preconception care which designed by CDC and WHO [2,4,6]. The possible explanation for this could be the service of preconception care was given less attention and the current focus of maternal health were prenatal and skilled birth services. In addition, substantial number of women were unaware about preconception care services that contributes for the lower uptake. Therefore, each stakeholders could work together to integrate and interlink with the routine maternal health services and encourage the awareness creation methods. Only 173(28.6%) of women were receive at least one items of preconception care service in their last pregnancy. The current finding higher than the study conducted in Debre birhan 13.4% of the women receive at least one component of preconception care and in other study conducted in Mekele 18.2% receive at least one services before pregnancy [17,24]. The justification for this may be difference in culture, perception to maternal health care and sociodemographic variation of the study participants.

The most common item received in the study area was taking micronutrient supplementation (iron and folate) (18.5%), which is lower than a report from a similar study conducted in Mekele city, northern Ethiopia (86.3%) [5],and southern Ethiopia(67.2%) [25] but higher than another study conducted in western Ethiopia (7.7%) [41]. This disparity could be difference in attention of maternal health service in different regions of the country Ethiopia. Even though, the country health care policy were the same, the implementation and approach in implementing may differs this could contribute for the variation. Furthermore, the current study was conducted in zonal town while Mekele city is regional town, which may have access of information and high level of health literacy of the study participants that could improve the use of PCC items.

The statistically significant factors for preconception care service use were maternal education, maternal age, husbands education, husbands occupation, wealth of households, distance from health facility, waiting time to the services, planning to pregnancy, previous ANC use, age at first pregnancy, previous PNC use, previous adverse pregnancy experience, knowledge on preconception care and attitude on preconception care. Maternal education were found to be a significant factor for receipt of preconception care service components. This finding also observed in previously conducted studies in Southern Ethiopia, Adet, Northern Ethiopia, Nigeria, China, Sri Lanka and Saudi Arabia [18,25–29]. This could be due to women with higher level of education have more access to information through reading, searching in internet due to strong information seeking ability which enables them to use more items of PCC. In addition education highly improves the health seeking behaviour of individuals and autonomous decision making ability [42,43]. Furthermore, education enables the women being responsive to the new health approaches and increase the desire to seek appropriate health care. In addition educated women have a capacity to obtained, process and understand the basic health information and services that enables a woman making positive decision to her own health [44].

Also, the current study showed that a significant association between maternal age and preconception care item service use. Compare to women age off 15–24, women’s age of 25–34 were 48% less likely use preconception care. Similar finding were observed in the study conducted in Brazil and China [22,23]. While This finding is not consistent with studies conducted in Debre birhan, Ethiopia [24]. The possible justification for this finding were younger age women could be lower daily activity and household task than older women.

Husbands’ educational status and working status were the other factors significantly associated with the receipt of preconception care components. Being merchant husbands’ occupation status were 23% more likely receipt preconception care items as compare with women’s husband had farmer. The justification could be due to ability of joint decision making regarding health of women and easily cover transportation cost of the women. The wealth of the household were positively associated with receipt of preconception care service components. The second, third, fourth and the fifth quantile of the households were 2.35,1.39,1.57 and 1.5 times more likely uses preconception care component as compare with the first(lowest) quantiles. Similar finding were observed in the study conducted in West Guji Zone, Oromia region Ethiopia [30]. The justification for this finding is due to economical empowered women can easily access the health facility. Wealth is component of having radio and TV that may disseminate information on preconception care could increase the awareness of the women. Distance from the health facility were one of the determinant factor for preconception care item use. Women who travel more than two kilometers were 44% less likely to use preconception care component as compare with counterpart. Similar finding were observed in the study conducted in southern Ethiopia [25]. Sustainable development goal woks for universal access of MNCH continuum of car and strongly advocate access of health care for all [45,46]. Reducing distance from health facility improve the health care utilization of the individuals [47]. The justification for this finding could be proximity to health facility improves the women use of maternal health service [48]. Geographical access to the health facility improves the health seeking behaviour of the women. This implies improving the access of the health facility could improve the receipt preconception care items.

Perceived waiting time to receive the services were the factor significantly associated with receipt preconception care items. Perceived waiting time less than 30minutes were 40% more likely to use preconception care items as compare with the counterpart. The justification of this finding was women had basic household tasks done on daily base so long waiting time to service may increase dropout from the services. In addition, the current study revealed a significant positive association between maternal knowledge of PCC and the use of PCC service items. Compared to those women who were knowledgeable, the chances of PCC item receipt were 47% less likely for poor knowledge women. This result is in line with the studies conducted in China, Saudi Arabia, Krea, Nepal, Nigeria, Southern, Ethiopia, Guji, Oromia region Ethiopia, and northern Ethiopia [23–25,29–32,38]. The justification of this could be more knowledgeable women had good understanding on advantage, place and content of service delivery which initiate more likely receive the recommended items of preconception care. As a result, multisectorial effort needed from the stakeholders in the town to improve women’s knowledge on PCC through behavioral change communication. In addition, this might be due to increase positive behaviour towards the action. This implies that improving the women’s knowledge could improve the utilization of preconception care.

Women with planned to pregnancy were the significant factor identified in the current study. Women who had not a plan to pregnancy were 55% less likely to use preconception care items as compare with counterpart. Similar finding was observed in the study conducted in Sri-Lanka, Iran and Adet, northern Ethiopia [18,28,33]. The justification of this finding could be women with planned pregnancy estimate and understand risks and maternal health effect on new coming baby. Furthermore, this could be due to increased health seeking behaviour of the women. An important requirement for preconception care was planning to pregnancy [49]. Therefore, unplanned pregnancy is the most integral issues that need to be addressed to increase utilization of preconception care. Previous maternal services such as ANC and PNC were the factors identified in the current study. Women who had never attended their previous ANC were 17% less likely to receipt PCC service items as compare with counterpart. This finding was similar with the study finding in Nigeria which showed that women who early attended ANC was more utilize preconception care [26]. This might be due to getting more information and the care of ANC might be increase motivation. Other finding indicated that women who never use of PNC were 33% less likely to receipt preconception care service items. The justification of this could be exposure to maternal health services enable them to understand, motivate, know and ask the additional services for better health. In this study, women who never experienced adverse pregnancy outcome were 68% less likely receipt preconception care service components as compare with counterpart. Similar finding was observed in study conducted in Mekelle, London, Belgium and Iran [17,34–36]. This might be due to increased self-responsibility and these women were more conscious on their next pregnancy. Age at first pregnancy were one of the factors identified in this study. Mothers age greater than 18 at first pregnancy were 2.58 times more likely received preconception care service items as compare with counterpart. The possible justification for this finding were the women with advanced age were understand them at risk so possibly they received PCC service items.

Furthermore, women’s attitude were one of the factors for preconception care practice. The women who had positive attitude for preconception care were 40% more likely to receipt preconception care service component as compare with counterpart. Similar finding was observed from the study conducted in Guji, Ethiopia [30]. The justification of this could be due to internal motivation could change to actual behaviour through removing of the barriers, searching information and understanding the benefits of preconception care use.

## Strength of study

Studying on both urban and rural areas could be able to elicit all factors that influence the utilization of preconception care.

### Limitation of the study

Cross sectional nature of the study that did not shows the actual cause effect relationship of the factors identified in the study.

## Conclusions

This study highlighted the low utilization of preconception car service items as compare with the recommended WHO and CDC component of care with higher disparities with different determinants. Age of women, educational status, husbands educational status, husbands occupation, wealth status, distance from the health facility, waiting time to get services, planning to pregnancy, age at first pregnancy, previous ANC use, Previous PNC use, adverse pregnancy experience, women’s knowledge of preconception care, and attitude on preconception care were factors identified in this study. This underscore of preconception care need a tailored intervention on women age 25–34 years old women with no formal education with living distant from the health facility and from the poor household category. In addition, advocative strategies on preconception care for women never experience adverse pregnancy outcome, not receipt PNC and ANC that may improve the use of preconception care items. The woreda and zonal health offices could work on access of health facility within two kilometers and integrate preconception care with maternal health services. The implementation strategies and policies could give an emphasis for a woman from poor household, distant from the health facility, never attended formal education and women’s husbands of a farmer through multipurpose approach, which address both medical and sociocultural issues faced by women.

## Supporting information

S1 Data (DTA)

S1 Appendix (DOCX)

S2 Appendix (DOCX)

## Abbreviations

ANC: Antenatal Care
AOR: Adjusted Odds Ratio
CDC: Centers for Disease Control and Prevention
CI: Confidence Interval
DM: Diabetes Mellitus
HF: Health Facility
HHs: Households
HIV: Human Immune Deficiency Virus
HTN: Hypertension
IRB: Institutional Review Board
MCH: Maternal and Child Health
OR: Odds Ratio
PCC: Preconception Care
WHO: World Health Organization
