# Supplementary material for: Preconception care utilization and associated factors among reproductive age women in Mizan-Aman town, Bench Sheko zone, Southwest Ethiopia, 2020. A content analysis
Source: PLoS One. 2022 Aug 19;17(8):e0273297. doi: 10.1371/journal.pone.0273297 (PMC9390911; doi:10.1371/journal.pone.0273297)
Supplement: S1 Appendix — (DOCX) [file pone.0273297.s002.docx]

## English version questionaries’

## Participant Information sheet and consent form

**Jimma University, Institute of Health, Faculty of public health, Department of population and family health**

**Project title:** Preconception Care and associated factors among reproductive age group women in Mizan-Aman town, Bench-Sheko Zone, Southwest, Ethiopia, 2020.

**Instruction:** please read a copy of the full informed consent or assent form to the respondent

**Introduction:**

Information sheet and consent form prepared for reproductive age women in Mizan-Aman town prior to the study to participant in this research project. The research group includes principal investigator, eight data collectors, and two supervisors.

**Purpose of the research:**

The aim of this study is to assess preconception care utilization and factors affecting the utilization and the information of this study will be useful for effective maternal and child health interventions. The study will be carried out for one month.

**Benefits, Risk and Discomfort:**

There might be slight discomfort to share some personal information and wasting your time (a maximum of 30 minutes). However, you may refuse to answer any of the questions if you feel uncomfortable. Your participation will help us to find more about factors affecting utilization of preconception care and this will help us to improve maternal preconception care in the town and other parts of the zone. There is no risk or direct benefit in the participating in this research.

**Incentive:**

We will not pay you for taking part in this study.

**Confidentiality:**

The information that we collected in this study will be kept confidential by using codes instead of any personal identifiers and is meant only for the purpose of the study.

**Right to refuse or withdrawal:**

You have the full right to refuse and have the right to discontinue the interview at any time, and refusing to participate will not affect anything you want.

**Who to contact:**

If you have any questions you may ask now or later. If you wish to ask questions later, you may contact: Melsew Setegn, Phone: +251938225950, E-mail: [melsewsetegn2010@gmail.com](mailto:melsewsetegn2010@gmail.com)

**Consent form** (Only for women age > 18 years old)

Hello! My name __________________and I am collecting data for the research being conducted by research team from Jimma University and Mizan-Tepi University. You are selected to be one of the participants from the study. This interview probably takes few minutes. I would like to assure you that all you tell during the interview will be strictly confidential and that information collected from you used only in scientific reports without any mentioning of personal information including your name. There is no harm or incentive for your participation. Information gathered from the study will be used to improve programs that promote maternal and child health.

Do you have any question? Can I proceed with the questions?

1. Yes ­­­­______ (thank you and continue) 2. No ____ (Thank you and stop)

**ASSENT** (Only for women age less than 18 years old)

Hello! My name is --------------------. I am collecting data for the research being conducted by research team from Jimma University and Mizan-Tepi University. They are doing research on Preconception Care and associated factors among reproductive age group women. Your wife/partner/child is scientifically been selected by chance to participate in this study. The findings of the study will be used for better planning and intervention of maternal health. Therefore, I request you kindly if you are volunteer that your wife/partner/child can participate in this study and respond to the questionnaire. The study involves no risk to your wife/partner/child. The information given is confidential. The interview by your wife/partner/child will take about few minutes to interview. Would you be willing for your wife/partner/child to participate?

1. Yes-------- (thank you and continue) 2. No---------- (Thank you and stop)

Name Kebele____________ Questioner code____________

Name of data collector__________________________sign____________date_______

Name of supervisor_____________________________________sign_____________date____

## English version questionaries’

**PART I: SOCIO-DEMOGRAPHIC CHARACTERISTICS OF THE RESPONDENTS**

| S/No | Question | Response | Skip |
| --- | --- | --- | --- |
| 101 | How old are you? | _______years old |  |
| 102 | What is your marital status? | 1. Married 2. Single 3. Separated 4. Widowed 5. Divorced 6. Others specify____________ |  |
| 103 | What is your religion? | 1. Orthodox 2. Muslim 3. Protestant 4. Catholic 5. Other(specify)_________ |  |
| 104 | Residence | 1. Urban 2. Peri-urban 3. Rural |  |
| 105 | What is your ethnicity? | 1. Banchi 2. Kefa 3. Amhara 4. Oromo 5. Welayita 6. Guraga 7. Tigra 8. Others specify___________ |  |
| 106 | What is your educational level? | 1. No formal education 2. Can read and write 3. Primary (1-8) 4. Secondary(9-12) 5. Tertiary(12+) |  |
| 107 | What is your main occupation? | 1. House wife 2. Farmer 3. Merchant 4. Government employee 5. Student 6. Private business 7. Daily worker 8. Other(specify)_________ |  |
| 108 | What is your husband educational level? | 1. No formal education 2. Can read and write 3. Primary (1-8) 4. Secondary(9-12) 5. Tertiary(12+) |  |
| 109 | What is the occupational status of housband? | 1. Farmer 2. Merchant 3. Government employee 4. Student 5. Private business 6. Daily worker 7. Other(specify)_________ |  |
| 110 | Family size | __________ |  |

**PART II: OBSTETRIC AND REPRODUCTIVE HISTORY**

| S/No | Question | Response | Skip |
| --- | --- | --- | --- |
| 201 | What was your age at your first marriage? | _______years |  |
| 202 | What was your age at your first pregnancy? | _______years |  |
| 203 | What is the total number of pregnancies in your life time (gravidity)? | _________ |  |
| 204 | What is the total number of live birth (parity)? | ___________ |  |
| 205 | Did you plan for your pregnancy? | 1. Yes 2. No |  |
| 206 | Have you ever had history of spontaneous abortion?(expulsion of fetus before 7months) | 1. Yes 2. No |  |
| 207 | Have you ever had history of still birth?(give birth to died fetus after its 7months) | 1. Yes 2. No |  |
| 208 | Have you ever had history of preterm birth?(give birth before 37weeks) | 1. Yes 2. No |  |
| 209 | Have you ever had history of congenital abnormality in your previous child? | 1. Yes 2. No |  |
| 210 | Have you ever had history of neonatal death? | 1. Yes 2. No |  |
| 211 | Have you ever gave birth to baby with the birth weight of <2.5kg(low birth weight) | 1. Yes 2. No |  |
| 212 | Did you have history of ANC visits for the index pregnancy? | 1. Yes 2. No | **If “No” go to 214** |
| 213 | How many times you visited ANC for the nearest pregnancy? | _________ |  |
| 214 | Did you have history of postnatal care for their index delivery? | 1. Yes 2. No | **If “No” go to 216** |
| 215 | How many times you visited PNC for the nearest delivery? | ________________ |  |
| 216 | Had you ever gave birth through cesarean section(operation)? | 1. Yes 2. No |  |
| 217 | Have you ever had history of contraceptive use? | 1. Yes 2. No | **If “No” go to 219** |
| 218 | What types of family planning you were using? | 1. IUCD  2. Implants  3. Injectable  4. Oral contraceptives  5. Condom  7. Emergency contraceptive  99. others specify_ |  |
|  |  |  |  |

**Part III:Questions to measure the women’s knowledge of preconception care**

| S/No | Questions | Response | Skip |
| --- | --- | --- | --- |
| 301 | Have you ever heard of care needed for the women before she becomes pregnant? | 1. Yes 2. No |  |
| 302 | What is the initial source of information? | 1. Health workers 2. School 3. In neighbors’ 4. Mass media 5. Family and friends   99.Others(specify)_ |  |
| 303 | Please tell me what should a woman do before getting pregnancy? | | |
|  | 1. HIV testing and counseling | 1 Yes 0. No |  |
|  | 2. Screening sexually transmitted infections | 1. Yes 0. No |  |
|  | 3. Screening for hypertension | 1. Yes 0. No |  |
|  | 4. Screening for diabetic mellitus | 1. Yes 0. No |  |
|  | 5. Screening for blood group | 1. Yes 0. No |  |
|  | 6. Screening for hepatitis b | 1. Yes 0. No |  |
|  | 7. Screening for low blood | 1. Yes 0. No |  |
|  | 8. Taking folic acid and multivitamins | 1. Yes 0. No |  |
|  | 9. Taking iron/ferrous | 1. Yes 0. No |  |
|  | 10. Immunization of tetanus vaccine/rubella | 1. Yes 0.No |  |
|  | 11. Screened for obesity? | 1. Yes 0. No |  |
|  | 12. Consulting health workers for advice | 1. Yes 0. No |  |
|  | 13. Having good nutrition/diet? | 1. Yes 0. No |  |
|  | 14. Avoiding /cessation of alcohol? | 1. Yes 0.No |  |
|  | 15. Avoiding /cessation of cigarette smoking? | 1. Yes 0.No |  |
|  | 16. Avoiding /cessation of chawing khat? | 1. Yes 0. No |  |
|  | 17. Discussion with the husband when to have child? | 1. Yes 0. No |  |
|  | 18.Stop/ remove family planning(if user) | 1. Yes 0. No |  |
|  | 19. Do you think husband’s heath condition matters for healthy conception? | 1. Yes 0.No |  |
|  | 20.Avoide illicit drugs | 1 Yes 0.No |  |
|  | 21.Stop over exercises | 1. Yes 0. No |  |
|  | 22.Away from Pesticides/insecticides chemicals | 1. Yes 0. No |  |
|  | 23.Away from exposure to occupational hazards | 1.Yes 0.No |  |
|  | 24.Stop caffeine drinking | 1.Yes 0.No |  |

**Part IV: questions on attitude of preconception care**

| s/no | Questions | Responses | Skip |
| --- | --- | --- | --- |
| 401 | Preconception care does not have any effect on pregnancy outcome | 1. Strongly disagree 2.disagree 3.neutral 4. Agree 5.strongly agree |  |
| 402 | Preconception care is an important health issue for women of child bearing age | 1. Strongly disagree 2.disagree 3.neutral 4. Agree 5.strongly agree |  |
| 403 | A dedicated clinic for preconception care is a luxury service | 1. Strongly disagree 2.disagree 3.neutral 4. Agree 5.strongly agree |  |
| 404 | A hospital setting is the best place to provide preconception care | 1. Strongly disagree 2.disagree 3.neutral 4. Agree 5.strongly agree |  |
| 405 | Preconception care is a high priority all mother to plan pregnancy | 1. Strongly disagree 2.disagree 3.neutral 4. Agree 5.strongly agree |  |
| 406 | I am not the most suitable person plan to get preconception care | 1. Strongly disagree 2.disagree 3.neutral 4. Agree 5.strongly agree |  |
| 407 | There is not enough time to plan to get a preconception care | 1. Strongly disagree 2.disagree 3.neutral 4. Agree 5.strongly agree |  |
| 408 | Health institutions exercise preconception care | 1. Strongly disagree 2.disagree 3.neutral 4. Agree 5.strongly agree |  |
| 409 | Do you think high-risk mothers only start preconception care when planned to pregnancy? | 1. Strongly disagree 2.disagree 3.neutral 4. Agree 5.strongly agree |  |
| 410 | History congenital anomalies only use preconception care | 1. Strongly disagree 2.disagree 3.neutral 4. Agree 5.strongly agree |  |
| 411 | Preconception care depends on health care providers’ willingness | 1. Strongly disagree 2.disagree 3.neutral 4. Agree 5.strongly agree |  |

**Part V: Questions on utilization of preconception care**

| S/no. | Question | Responses | Skip |
| --- | --- | --- | --- |
| 500 | Did you take folic acid for the sake of becoming pregnant? | 1.Yes 0.No |  |
| 501 | Did you take iron/ ferrous for the sake of becoming pregnant? | 1. Yes 0. No |  |
| 502 | Did you prepare diet from different cereals for the sake of becoming pregnant? | 1.Yes 0.No |  |
| 503 | Did you take extra meal for the sake of becoming pregnant? | 1.Yes 0.No |  |
| 504 | Did you check your weight for the sake of becoming pregnant? | 1.Yes 0.No | **If No go to 506** |
| 505 | What did you do after you checked your weight for the sake of becoming pregnant? | 1.increase weight  2.decrease weight  3.nether increased or decreased |  |
| 506 | Did you screen for HIV/AIDS for the sake of becoming pregnant? | 1.Yes 0.No |  |
| 507 | Did you screen for STI for the sake of becoming pregnant? | 1.Yes 0.No |  |
| 508 | Did you screen for diabetes mellitus for the sake of becoming pregnant? | 1.Yes 0.No |  |
| 509 | Did you screen for hypertension for the sake of becoming pregnant? | 1.Yes 0.No |  |
| 510 | Did you screen for low blood (anemia) for the sake of becoming pregnant? | 1.Yes 0.No |  |
| 511 | Did you screen for blood group for the sake of becoming pregnant? | 1.Yes 0.No |  |
| 512 | Did you screen for hepatitis for the sake of becoming pregnant? | 1. Yes 0. No |  |
| 513 | Did you take tetanus vaccine for the sake of becoming pregnant? | 1.Yes 0.No |  |
| 514 | Had you ever smoke in your life time? | 1.Yes 0.No | **If No go to 517** |
| 515 | Did you stop or reduced smoking? | 1.Yes 0.No |  |
| 516 | For what reasons you stopped/reduced smoking? | 1.for the sake of becoming pregnant  2.other(specify)______ |  |
| 517 | Had you drinking alcohol in your life? | 1.Yes 0.No | **If No go to 520** |
| 518 | Did you stopped/reduced drinking alcohol? | 1.Yes 0.No |  |
| 519 | For what reasons did you stopped/reduced drinking alcohol? | 1.for the sake of becoming pregnant  2.other(specify)______ |  |
| 520 | Did you chewing khat in your life? | 1.Yes 0.No | **If No go to 523** |
| 521 | Did you stopped/reduced chewing khat | 1.Yes 0.No |  |
| 522 | For what reasons did you stopped/reduced chewing khat | 1.for the sake of becoming pregnant  2.other(specify)______ |  |
| 523 | Did you consult anyone for advice for the sake of becoming pregnant? | 1.Yes 0.No |  |
| 524 | Did your husband screened for any disease for the sake of your becoming pregnant? | 1.Yes 0.No |  |
| 525 | Did you stopped or removed family planning (if user)? | 1.Yes 0.No |  |

**Part VI: Pre-existing Medical illness**

| S/no | Questions | Responses | Skip |
| --- | --- | --- | --- |
| 601 | Do you have any illness for which you have follow up at the health facility? | 1. Yes 2. No | **If no go to 701** |
| 602 | Previous Epilepsy | 1. Yes 0. No |  |
|  | Previous Hypertension | 1. Yes 0. No |  |
|  | History of Diabetes mellitus | 1. Yes 0. No |  |
|  | Previous Anemia History of | 1. Yes 0.No |  |
|  | Asthma History of cardiac | 1.Yes 0.No |  |
|  | problems History of Renal disease | 1. Yes 0.No |  |
|  | Previous HIV/AIDS | 1. Yes 0.No |  |
|  | Others Specify | ________________ |  |
| 603 | Did the health provider give any care for the sake of pregnancy? | 1. Yes 2. No |  |

**Part VII: Health facility related questions**

| S/no | Questions | Responses | Skip |
| --- | --- | --- | --- |
| 701 | Approximately how many minutes/kilometer does it take to reach the nearby health facility(health center, hospital) | _________minutes  __________kilometers |  |
| 702 | What is the means of transport to reach health facility | 1.private care 2.ambulance 3 motor cycle 4.public bus 5 foot  99.other(specify) |  |
| 703 | Did you get the service for the sake of your healthy pregnancy whenever you went to the health facility? | 1. Yes 2. No(specify)______ 3. Not applicable |  |
| 704 | Did you pay in order to get services for the sake of becoming pregnant? | 1. Yes 2. No | **If no go to 706** |
| 705 | How affordable the payment is to you? | 1. Expensive 2. Fair 3. Cheap 4. Free |  |
| 706 | Were you using health insurance before you become pregnant? | 1. Yes 2. No |  |
| 707 | Whenever you went to the health facility for any services did your health care provider tell you about the care needed for the women she become pregnant? | 1. Yes 2. No |  |
| 708 | Availability of adequate laboratory service | 1. Yes 2. No   3. I do not know |  |
| 709 | Availability of adequate medication | 1. Yes 2. No 3. I do not know |  |
| 710 | Availability of PCC unit | 1. Yes 2. No |  |
| 711 | Autonomy to maternal health service | 1. By joint decision 2. By self-decision 3. By husband decision |  |
| 712 | From whom you receive health care access assistance | 1. From my husband 2. From relatives 3. From families 4. From neighbors |  |
| 713 | Availability of Guideline | 1. Yes 2. No 3. I do not know |  |
| 714 | Distance from the health facility(How much far away) | ____________ |  |
| 715 | Perceived time spent to get services | ___________________ |  |

**Part VII: wealth of the household**

| S/no | Questions | Responses | Skip |
| --- | --- | --- | --- |
| 801 | What is the main source of water for members of your household? | 1. Pipe water 2. Protected well 3. Unprotected well 4. Others(specify)____ | **Multiple answer is possible** |
| 802 | What kind of toilet facility do members of your household usually use? | 1. Ventilated improved pit latrine (VIP) 2. Pit latrine with slap 3. Pit latrine without slap/open pit 4. No facility/bush/field   99.Other(specify)___ |  |
| 803 | Does your household have: | 1. bed 2.bicycle 3.motorcycle  4. care 5.tables  6.chair 7.watch  8.radio 9.mobile telephone  10.electricity  11.generator 12.solar  13.television 13.sofa | **Multiple answer is possible** |
| 804 | Do you have separate room that is used as kitchen | 1.yes  0.no |  |
| 805 | Main material of the floor | 1.earth  2.wooden  3.Cement  99. Other(specify)_____ | **Record by observation** |
| 806 | Main material of the roof | 1. Grass 2. Corrugated iron sheet 3. Cement   99.Others(specify)____ | **Record by observation** |
| 807 | Main material of the walls | 1. Wooden or mud 2. Wood/sticks and cement 3. Bricks or shekila   99.Other(specify) | **Record by observation** |
| 808 | How many household members are sleeping in one room? | ________________ |  |
| 809 | Does any member of the household own land that can be used for agricultures? | 1. Yes 2. No |  |
| 810 | Does any member of the household own land that can be used for coffee? | 1. Yes 2. No |  |
| 811 | Does this household own any livestock, herd, or farm animals? | 1. Yes 2. No | **If no go to No.813** |
| 812 | How many of the following animals do this household own? | 1. Milk cows_____ 2. Oxen or bull____ 3. Horses______ 4. Mules_____ 5. Sheep_____ 6. Chicken____   99.Others_____ |  |
| 813 | Do you have bank account? | 1. Yes 0.no |  |
| 814 | If the response of Q813 is yes how many birr do you have on the account? | ______ETB |  |

**Thank you!!!**

Thank you!!!
