# Supplementary material for: Preconception care utilization and associated factors among reproductive age women in Mizan-Aman town, Bench Sheko zone, Southwest Ethiopia, 2020. A content analysis
Source: PLoS One. 2022 Aug 19;17(8):e0273297. doi: 10.1371/journal.pone.0273297 (PMC9390911; doi:10.1371/journal.pone.0273297)
Supplement: S2 Appendix — (DOCX) [file pone.0273297.s003.docx]

## Amharic version of the data collection tool

አባሪ I: የተሳትፎመረጃወረቀትእናስምምነትቅጽ

ጅማዩኒቨርስቲ፣የጤናሳይንስተቋም፣የህብረተሰብጤናፋኩልቲ፣የህዝብቁጥርእናየቤተሰብጤናት/ት ክፍል

**የፕሮጀክትርዕስ-**ቅድመእርዝናእንክብካቤአጠቃቀምእናተያያዥነትያላቸውንምክንያቶችለመገምገም ሚዛን- አማንከተማበደቡብምዕራብኢትዮጵያ 2020 እ.ኤ.አ

**የድርጅቱስም**- **ጅማዩኒቨርስቲ**

**መመሪያ**: እባክዎንሙሉየመረጃስምምነትቅጽንያንብቡለተሳታፈው

**መግቢያ**

በዚህየምርምርፕሮጄክትውስጥተሳታፊከመሆናቸውበፊትበሚዛን-አማንከተማበመውለድዕድሜክልል ያሉ ሴቶችየመረጃልውውጥእናየስምምነትቅጽተዘጋጅቷል፡፡የጥናቱቡድንየጥናቱባለቤት፣ስምንትየመረጃአሰባሳቢዎችእናሁለትተቆጣጣሪዎችንያጠቃልላል፡፡

**የጥናቱዓላማ**

የዚህጥናትዓላማ- ቅድመእርዝናእንክብካቤአጠቃቀምእናአጠቃቀሙንየሚመለከቱጉዳዮችንለመገምገምበሚዛን-አማንከተማበመውለድዕድሜክልል ያሉ ሴቶችበደቡብምዕራብኢትዮጵያ 2020፡፡ የዚህጥናትመረጃውጤታማየእናቶችናየህፃናትጤናለማሻሻልጠቃሚነው፡፡ጥናቱለአንድወርይካሄዳል፡፡

**ጥቅማጥሞችእናስጋት**

የእርስዎተሳትፎቅድመእርዝናናየቅድመወሊድእንክብካቤአጠቃቀምንስለሚመለከቱጉዳዮችየበለጠእንድናውቅይረዳናልእናምይህበከተሞችእናበሌሎችየዞንክፍሎችየእናቶችንቅድመእርዝናጥንቃቄንለማሻሻልይረዳናል፡፡በዚህምርምርውስጥመሳተፍአደጋወይምቀጥተኛጥቅምየለም፡፡

**ማበረታቻ**

በዚህጥናትውስጥበመሳተፍአንከፍልዎትም፡፡

**ምስጢራዊነት**

በዚህጥናትውስጥየሰበሰብነውመረጃከማንኛውምየግልመለያዎችበሚስጥርይጠበቃልእናምለጥናቱዓላማብቻነው፡፡

**የመቃወምወይምየመተውመብት**

እምቢታምየመቃወምሙሉመብትእናበማንኛውምጊዜቃለመጠይቅዎንየማቋረጥመብትአልዎት፣እናለመሳተፍእምቢበማለቶየሚፈልጉትንነገርአይጎዳውም፡፡

**ማንንመገናኘትእንዳለብዎት**

መጠየቅየሚፈልጉትነገርካለአሁንወይምበኋላመጠየቅይችላሉ፡፡ጥያቄዎችንበኋላመጠየቅከፈለጉ, መልሰውሰጠኝንሊጠይቁይችላሉ፤ስልክ: +251938225950, ኢሜል: melsewsetegn2010@gmail.com

**ስምምነት (ለ 18 አመት እና ከዚያ በላይ ለሆኑ ሴቶች ብቻ)**

ጤናይስጥልኝ: ስሜ __________________እባላለሁ; እናየጅማዩኒቨርስቲ ለሚካሄደውጥናትመረጃእሰበስባለሁ።ቅድመእርዝናእንክብካቤአጠቃቀምእናአጠቃቀሙንየሚመለከቱጉዳዮችንለመገምገምበመውለድዕድሜያቸው 15-49 ሴቶችመካከልያሉተጓዳኝነጥቦችንምርምርእያደረገነው፡፡እርስዎከጥናቱተሳታፊዎችእንዲሆኑተመርጠዋል፡፡ይህቃለመጠይቅምናልባትጥቂትደቂቃዎችንይወስዳል፡፡በቃለ-መጠይቁወቅትየሚናገሩትነገርበሙሉበሚስጢርየተጠበቀእንደሚሆንናስምዎንምጨምሮየግልመረጃዎንሳይጠቅሱበሳይንሳዊሪፖርቶችውስጥብቻያገለገሉመሆናቸውንማረጋገጥእፈልጋለሁ፡፡ለተሳትፎዎምንምጉዳትወይምማበረታቻየለም፡፡ከጥናቱየተሰበሰበውመረጃየእናትንእናየህፃናትንጤናየሚያበረታቱፕሮግራሞችንለማሻሻልጥቅምላይይውላል፡፡ስለዚህጥናትማንኛውንምጥያቄካልዎትእኔንወይምዋናተመርማሪውንመጠየቅይችላሉ፡፡የአቶመልሰውሰጠኝስልክቁጥር +251938225950 ወይምበኢሜል melsewsetegn2010@gmail.com በመጠቀምመጠየቅይችላሉ

ጥያቄአለዎት? በጥያቄዎቹመቀጠልእችላለሁን?

1.አ ዎ_______(እናመሰግናለንእና ቀጥል) 2. አይ_______ (እናመሰግናለንእና አቁም)

**በተጠያቂዋ ምትክ ከአሳዳሪ የሚወሰድ ስምምነት (ከ 18 ዓመት በታች ለሆኑ ወጣት ሴቶች ብቻ)**

ጤናይስጥልኝ: ስሜ __________________እባላለሁ; እናየጅማዩኒቨርስቲለሚካሄደውጥናትመረጃእሰበስባለሁ።ቅድመእርዝናእንክብካቤአጠቃቀምእናአጠቃቀሙንየሚመለከቱጉዳዮችንለመገምገምበመውለድዕድሜያቸው 15-49 ሴቶችመካከልያሉተጓዳኝነጥቦችንምርምርእያደረገነው፡፡ምስትዎ /ፍቅረኛዎ /ልጅዎ በዚህ ጥናት ለመሳተፍ በሳይንሳዊ መንገድ የተመረጠች ሲሆን የጥናቱ ግኝት ከላይ ከተጠቀሰው መረጃ እንደሰሙት የእናትንእናየህፃናትንጤናየሚያበረታቱፕሮግራሞችንለማሻሻልጥቅምላይይውላል፡፡ስለዚህ ባለቤትዎ /ፍቅረኛዎ /ልጅዎ በዚህ ጥናት መሳተፍ እና ለመጠይቁ ምላሽ መስጠት እንዲችሉ በትህትና እጠይቃለሁ፡፡ ጥናቱ ለሚስትዎ / ለአጋርዎ / ለልጅዎ ምንም አደጋ የለውም፡፡ የሚሰጡን መረጃ ሚስጢራዊ ነው፡፡ ይህቃለመጠይቅምናልባትጥቂትደቂቃዎችንይወስዳል፡፡ ስለ ሚስትዎ / ለጓደኛዎ / ልጅዎ እንዲሳተፉ ፈቃደኛ ይሆናሉ?

1. አዎ -------- 2. አይ ---------- አመሰግናለሁ!

ቀበሌስም________ _________ የጥያቄኮድ _______________

የመረጃሰብሳቢውስም**­­­­­­­­­­­­**ፊርማ__________________ቀን­­­­­­­­­­­­­­­­­_________________

ተቆጣጣሪውስም__________________________ፊርማ______________________ቀን___________

**ክፍል I-አጠቃላይማህበራዊመረጃ**

| ተራቁጥር | ጥያቄ | መልስ | **ወደ… ይሂዱ** |
| --- | --- | --- | --- |
| 101 | ዕድሜዎስንትነው? | ------------አመት |  |
| 102 | የጋብቻሁኔታዎምንድነው? | 1 ያገባች  2 ያላገባች  3 የተለያየች  4 ባሏየሞተባት  5 የተፋታች  6 ሌሎችይጥቀሱ ____ |  |
| 103 | ሃይማኖትዎምንድነው? | 1. ኦርቶዶክስ  2. ሙስሊም  3. ፕሮቴስታንት  4. ካቶሊክ  5. ሌላ (ይግለጹ) _________ |  |
| 104 | ቋሚመኖሪያዎየትነው? | 1. ከተማ 2. ከተማ አባ 3.ገጠር |  |
| 105 | ብሄርዎምንድነው? | 1. ቤንች 2. ከፋ  3. አማራ 4. ኦሮሞ  5. ወላይታ 6. ጉራጋ  7. ትግሬ  8. ሌሎችይጥቀሱ ___________ |  |
| 106 | የትምህርትደረጃዎምንድነው? | 1. ያልተማረች 2. ማንበብና መጣፍ ብቻ   3. የመጀመሪያደረጃ (1-8)  4. ሁለተኛ (9-12)  5.ከሁለተኛደረጃበላይ (12+) |  |
| 107 | ዋናሥራዎትምንድንነው? | 1. የቤትእመቤት 2. ገበሬ  3. ነጋዴ 4. የመንግስትሰራተኛ  5. ተማሪ 6. የግልሰራተኛ  7. የቀንሰራተኛ  8. ሌላ (ይግለጹ) _____ |  |
| 1ዐ8 | የባልሽየትምህርትደረጃምንድነው? | 1. ያልተማረ  2. ማንበብና መጣፍ ብቻ  3. የመጀመሪያደረጃ (1-8)  4. ሁለተኛ (9-12)  5. ከሁለተኛደረጃበላይ (12+) |  |
| 109 | የባልሽ ዋናሥራዎትምንድንነው? | 2. ገበሬ  3. ነጋዴ 4. የመንግስትሰራተኛ  5. ተማሪ 6. የግልሰራተኛ  7. የቀንሰራተኛ  8. ሌላ (ይግለጹ) _____ |  |
| 11ዐ | የቤተሰብመጠን | ­­­­____________ |  |

**ክፍል II: - የሥነተዋልዶመረጃ**

| ተራቁጥር | ጥያቄ | መልስ | **ወደ… ይሂዱ** |
| --- | --- | --- | --- |
| 201 | በመጀመሪያ ጋብቻዎ ዕድሜዎ ምን ያህል ነበር | ________ዓመታት |  |
| 202 | በመጀመሪያው የእርግዝና ወቅትዎ ዕድሜዎ ስንት ነበር? | __________ዓመታት |  |
| 203 | በሕይወትዎጊዜውስጥአጠቃላይየእርግዝናብዛትስንትነው? | ­­­­----------- |  |
| 204 | እስካሁንስንትልጅወለድሽ? | -----------ልጅ |  |
| 205 | ለመውለድ አቅደሽታውቂያለሽ? | 1. አዎ 0. አይድለም |  |
| 206 | ማስወረድአጋጥሞዎትያውቃሉ (ከ 7 ወርበፊትፅንስማስወረድ) | 1. አዎ 0. አይድለም |  |
| 207 | እስካሁንበሕይወትየለሌልጅወለድሽታውቂያለሽ? | 1. አዎ 0. አይድለም |  |
| 208 | ከ 37 ሳምንትበፊትየተወለደልጅነበረሽ? | 1. አዎ 0. አይድለም |  |
| 209 | በልጅዎውስጥአካለመጓደልታሪክአጋጥመውያውቃሉ? | 1. አዎ 0. አይድለም |  |
| 210 | ተወለዶበአንድወርየሞትህፃንነበረሽ? | 1. አዎ 0. አይድለም |  |
| 211 | ክብደቱ 2.5KG በታችየሆነ/ችልጅወለድሽታውቂያለሽ? | 1. አዎ 0. አይድለም |  |
| 212 | ለአለፈውእርግዝናየቅድመወሊድምርመራ አድርገውነበር? | 1. አዎ 0. አይድለም | **አይድለምከሆነወደ 214 ይሂዱ** |
| 213 | 212 መልስአዎከሆነስንትግዜ? | --------- |  |
| 214 | የድሕረ ወለድ ምርመራአድርገውያውቃሉ? | 1. አዎ 0. አይድለም | **አይድለምከሆነወደ 216 ይሂዱ** |
| 215 | 214 መልስአዎከሆነስንትግዜ? | _________ |  |
| 216 | በቀዶ ጥገና ወልደው ያውቃሉ? | 1. አዎ 0.አይድለም |  |
| 217 | የእርግዝናመከላከያተጠቅመሽታውቂያለሽ? | 1. አዎ 0. አይድለም | **አይድለምከሆነወደ 301 ይሂዱ** |
| 218 | 217 መልስአዎከሆነየሚጠቀሙትየቤተሰብዕቅድምንዓይነትነበር? | 1. በማህጸንየሚቀመጥ (IUCD)  2. በግንድየሚቀበር (Implants)  3. በመርፌየሚሰጥየወሊድመከላከያ  4. የሚወጥየወሊድመከላከያእንክብል  5. ኮንዶም  6. ከወስብበኋላየሚወጥ (Postpill)  99. ሌላደግሞይጠቀስ………………… _____ |  |

**ክፍል III: ስለቅድመ-እርግዝናእንክብካቤአገልግሎትግንዛቤ**

| ተራቁጥር | ጥያቄ | መልስ | **ወደ… ይሂዱ** |
| --- | --- | --- | --- |
| 301 | ቅድመእርግዝናእንክብካቤሰምተሽታውቂያለሽ?(ሴቶችከመፀነስበፊትየሚያደርጉትማንኛውምነገር) | 1. አዎ 0. አይድለም |  |
| 302 | 301 መልስአዎከሆነከየትሰሙ? | 1. የጤናሰራተኞች  2. ትምህርትቤት  3. በጎረቤቶች  4. መገናኛብዙሃን  5. ቤተሰብእናጓደኞች  99. ሌሎች (ይጥቀሱ)___ |  |
| 303 | እባክሽአንዲትሴትምን አይነት ቅድመእርግዝናእንክብካቤማድረግእንዳለባትንገረኝ? | | |
|  | 1. የኤች.አይ.ቪምርመራእናምክር | 1. አዎ 0. አይድለም |  |
|  | 2 የደምግፊትምርመራ | 1. አዎ 0. አይድለም |  |
|  | 3 የአባላዛርምርመራ | 1. አዎ 0. አይድለም |  |
|  | 4 የስኳርበሽታምርመራ | 1. አዎ 0. አይድለም |  |
|  | 5. የደምአይነትምርመራ | 1. አዎ 0.አይድለም |  |
|  | 6 የጉበትበሽታምርመራ | 1. አዎ 0.አይድለም |  |
|  | 7 ለደምማነስምርመራ | 1. አዎ 0. አይድለም |  |
|  | 8 ፎሊክአሲድመውሰድ | 1. አዎ 0. አይድለም |  |
|  | 9 የደም ማነስ መድህኒይት (አይረን)መውሰድ | 1. አዎ 0. አይድለም |  |
|  | 10. የመጋጋ ቆልፍ (tetanus) ክትባትመውሰድ | 1. አዎ 0. አይድለም |  |
|  | 11. የክብደትመጠንምርመራ | 1. አዎ 0. አይድለም |  |
|  | 12. ምክርለማግኘትየጤናሠራተኞችንማማከር | 1. አዎ 0. አይድለም |  |
|  | 13. ጥሩአመጋገብ / አመጋገብአለዎት? | 1. አዎ 0.አይድለም |  |
|  | 14. አልኮልንመቀነስ / ማቆም? | 1. አዎ 0. አይድለም |  |
|  | 15. ሲጋራማጨስንማስቀረት / ማቆም? | 1. አዎ 0. አይድለም |  |
|  | 16. ጫትመቃምአቁመዋል / ቀንሰዋል | 1. አዎ 0. አይድለም |  |
|  | 17. መቼልጅመውለድእንዳለበትከባለቤቱጋርየሚደረግውይይት? | 1. አዎ 0. አይድለም |  |
|  | 18. የቤተሰብንእቅድያውጡ / ያስወግዱ (ተጠቃሚውከሆነ) | 1. አዎ 0. አይድለም |  |
|  | 19. የባለቤቶየጤናሁኔታበእርግዝናውላይተጥኖያመጣል | 1. አዎ 0. አይድለም |  |
|  | 20. የተከለከለ መድህኒይት አለመጠቀም | 1. አዎ 0. አይድለም |  |
|  | 21.ከመጠን ያለፈ እንቅስቃሴ አለመራት | 1. አዎ 0. አይድለም |  |
|  | 22.ከተባይ መድህኒይት መራቅ | 1. አዎ 0. አይድለም |  |
|  | 23.አደጋ ከሚያጋልጥ ስራ መራቅ | 1. አዎ 0. አይድለም |  |
|  | 24. አነቃቂ መድህኒይት አለመጠቀም | 1. አዎ 0. አይድለም |  |

**ክፍልIV :ቅድመእርግዝናእንክብካቤ ዝንባሌ**

| ተራቁጥር | ጥያቄ | መልስ | **ወደ… ይሂዱ** |
| --- | --- | --- | --- |
| 401 | ቅድመእርግዝናእንክብካቤማድረግበእርግዝናውጤትላይምንምተፅእኖየለውም | 1. በጣምአልስማማም 2. አልስማማም 3. ምንም 4. እስማማለሁ 5. በጣምእስማማለሁ |  |
| 402 | ቅድመእርግዝናእንክብካቤማድረግለእቶችናሕፃናትጤና አስፈላጊነው | 1. በጣምአልስማማም 2. አልስማማም 3. ምንም 4. እስማማለሁ 5. በጣምእስማማለሁ |  |
| 403 | ቅድመእርዝናእንክብካቤክሊኒክአገልግሎትየቅንጦት ነው | 1. በጣምአልስማማም 2. አልስማማም 3. ምንም 4. እስማማለሁ 5. በጣምእስማማለሁ |  |
| 404 | ቅድመእርግዝናእንክብካቤለመስጠትየሆስፒታልአቀማመጥበጣምጥሩ ቦታነው | 1. በጣምአልስማማም 2. አልስማማም 3. ምንም 4. እስማማለሁ 5. በጣምእስማማለሁ |  |
| 405 | ቅድመእርግዝናእንክብካቤለማርገዝእቅድ ላላቸው እናቶችቅድሚያ እርግዝናበጣምአስፈላጊጉዳይነው | 1. በጣምአልስማማም 2. አልስማማም 3. ምንም 4. እስማማለሁ 5. በጣምእስማማለሁ |  |
| 406 | ቅድመእርግዝናእንክብካቤለእኔ አስፈላጊአድለም | 1. በጣምአልስማማም 2. አልስማማም 3. ምንም 4. እስማማለሁ 5. በጣምእስማማለሁ |  |
| 407 | ቅድመእርግዝናእንክብካቤለማድረግበቂጊዜየለም | 1. በጣምአልስማማም 2. አልስማማም 3. ምንም 4. እስማማለሁ 5. በጣምእስማማለሁ |  |
| 408 | የጤናተቋማትቅድመእርዝናእንክብካቤያካሂዳሉ | 1. በጣምአልስማማም 2. አልስማማም 3. ምንም 4. እስማማለሁ 5. በጣምእስማማለሁ |  |
| 409 | ቅድመእርግዝናእንክብካቤለአደጋየተጋለጡእናቶችበእርግዝናወቅትየታቀደየቅድመወሊድእንክብካቤየሚጀምሩትይመስልዎታል? | 1. በጣምአልስማማም 2. አልስማማም 3. ምንም 4. እስማማለሁ 5. በጣምእስማማለሁ |  |
| 410 | አካለ የሌለው ህፃን ለወለደች እናትየሚጠቀሙትቅድመ-ጥንቃቄንብቻነው | 1. በጣምአልስማማም 2. አልስማማም 3. ምንም 4. እስማማለሁ 5. በጣምእስማማለሁ |  |
| 411 | ቅድመእርግዝናእንክብካቤበጤናሠራተኞችፈቃደኛነትላይየተመሠረተነው | 1. በጣምአልስማማም 2. አልስማማም 3. ምንም 4. እስማማለሁ 5. በጣምእስማማለሁ |  |

**ክፍልV :ቅድመእርግዝናእንክብካቤአጠቃቀም**

| ተራቁጥር | ጥያቄ | መልስ | **ወደ… ይሂዱ** |
| --- | --- | --- | --- |
| 500 | እርጉዝለመሆንሲሉፎሊክአሲድ/ቪታሚንወስደዋል? | 1. አዎ 0. አይድለም |  |
| 501 | እርጉዝለመሆንሲሉየደም ማነስ መድህኒይት (አረን)ወስደዋልን? | 1. አዎ 0. አይድለም |  |
| 502 | እርጉዝለመሆንሲሉከተለያዩእህሎችውስጥምግብያዘጋጁነበር? | 1. አዎ 0. አይድለም |  |
| 503 | እርጉዝለመሆንሲሉተጨማሪምግብወስደዋል? | 1. አዎ 0. አይድለም |  |
| 504 | እርጉዝለመሆንሲሉክብደትዎንተመዝነዋል?? | 1. አዎ 0. አይድለም | **አይድለምከሆነወደ 506 ይሂዱ** |
| 505 | ለማርገዝሲሉክብደትዎንከተመዝንሽበኋላምንአደረጉ? | 1.ክብደትጨምርሽ  2. ክብደትቀነስሽ  3. አልጨርሽምወይምአልቀነስሽም |  |
| 506 | ለማርገዝብለሽየኤችአይቪ / ኤድስንምርመራአደርገሻል? | 1. አዎ 0. አይድለም |  |
| 507 | ለማርገዝብለሽየአባላዛር ምርመራአደርገሻል? | 1. አዎ 0. አይድለም |  |
| 508 | ለማርገዝብለሽየስኳርበሽታምርመራአደርገሻል | 1. አዎ 0. አይድለም |  |
| 509 | ለማርገዝብለሽየደምግፊትምርመራአደርገሻል? | 1. አዎ 0. አይድለም |  |
| 510 | ለማርገዝብለሽየደምማነስምርመራአደርገሻል? | 1. አዎ 0. አይድለም |  |
| 511 | ለማርገዝብለሽየደምአይትምርመራአደርገሻል? | 1. አዎ 0. አይድለም |  |
| 512 | ለማርገዝብለሽየጉበትበሽታምርመራአደርገሻል? | 1. አዎ 0. አይድለም |  |
| 513 | ለማርገዝብለሽየመጋጋ ቆልፍክትባትወስደዋል? | 1. አዎ 0. አይድለም |  |
| 514 | በሕይወትዎዘመንሽሲጋራአጭሰሽታውቂያለሽ? | 1. አዎ 0. አይድለም | **አይድለምከሆነወደ 517 ይሂዱ** |
| 515 | ሲጋራአቁመዋልወይምቀንሰዋል? | 1. አዎ 0. አይድለም |  |
| 516 | ሲጋራአቁመውወይምቀንሰውከሆነለምን? | 1. ለማርገዝብየ  2. ሌላ (ይግለጹ) ______ |  |
| 517 | በሕይወትዎዘመንሽአልኮልጠጥተሽታውቂያለሽ? | 1. አዎ 0. አይድለም | **አይድለምከሆነወደ 520 ይሂዱ** |
| 518 | አልኮልአቁመዋልወይምቀንሰዋል? | 1. አዎ 0. አይድለም |  |
| 519 | አልኮልአቁመውወይምቀንሰውከሆነለምን? | 1 ለማርገዝብየ  2. ሌላ (ይግለጹ) ______ |  |
| 520 | በሕይወትዎዘመንሽጫትቅመሽታውቂያለሽ? | 1. አዎ 0. አይድለም | **አይድለምከሆነወደ 523 ይሂዱ** |
| 521 | ጫትመቃምአቁመዋልወይምቀንሰዋል? | 1. አዎ 0. አይድለም |  |
| 522 | ጫትመቃምአቁመውወይምቀንሰውከሆነለምን? | 1 ለማርገዝብየ  2. ሌላ (ይግለጹ) ______ |  |
| 523 | እርጉዝለመሆንሲሉማንኛውንም ሰውምክርያማክሩነበር? | 1. አዎ 0. አይድለም |  |
| 524 | ለእርግዝናሽብሎባልሽማንኛውንምበሽታተመርምሯል? | 1. አዎ 0. አይድለም |  |
| 525 | እርጉዝለመሆንሲሉየቤተሰብዕቅድአቁመዋልወይምአስወግደዋል (ተጠቃሚከሆነ)? | 1. አዎ 0. አይድለም |  |

**ክፍል VI: እርዝናበፊትስነበረበሽታወች/የጤናችግሮች**

| ተራቁጥር | ጥያቄ | መልስ | **ወደ… ይሂዱ** |
| --- | --- | --- | --- |
| 601 | በጤናተቋሙውስጥክትትልየሚያደርጉበትበሽታአለ? | 1. አዎ 0. አይድለም | **አይድለምከሆነወደ 701 ይሂዱ** |
| 602 | 1. ካሁንበፊትደምግፊትታምሚያለሁ? | 1. አዎ 0. አይድለም |  |
|  | 1. የስኳርበሽታአለብሽ? | 1. አዎ 0. አይድለም |  |
|  | 1. ካሁንበፊትየደምማነስይዘኝያውቃል? | 1. አዎ 0. አይድለም |  |
|  | 1. የአስምበሽታአለብሽ? | 1. አዎ 0. አይድለም |  |
|  | 1. የልብበሽታአለብሽ? | 1. አዎ 0. አይድለም |  |
|  | 1. የነቀርሳበሽታ (ካንሰር) አለብሽ? | 1. አዎ 0. አይድለም |  |
|  | 1. የኩላሊትበሽታአለብሽ? | 1. አዎ 0. አይድለም |  |
|  | 99. ሌላካለይጠቀስ_______ |  |  |
| 603 | የጤናአገልግሎትሰጪውለእርግዝናሲባልምንምዓይነትእንክብካቤአልሰጠም? | 1. አዎ 0. አይድለም |  |

**ክፍል VII: ከጤናተቋማትጋርየሚዛመዱጥያቄዎች**

| ተራቁጥር | ጥያቄ | መልስ | **ወደ… ይሂዱ** |
| --- | --- | --- | --- |
| 700 | በቂ መድሃኒት አለ | 1 አዎ 2 አይ 3 አላውቅም |  |
| 701 | የእናቶች ጤና አገልግሎት ራስን በራስ የማስተዳደር | 1 በጋራ ውሳኔ 2 በራስ የመወሰን 3 በባል ውሳኔ |  |
| 702 | በቂ የላብራቶሪ አገልግሎት መገኘት | 1 አዎ 2 አይ 3 አላውቅም |  |
| 703 | ከማን የጤና እንክብካቤ መዳረሻ እርዳታ ያገኛሉ | 1 ከባለቤቴ 2 ከዘመዶች 3 ከቤተሰብ 4 ከጎረቤቶች |  |
| 704 | የመመሪያው መገኘት | 1 አዎ 2 አይ 3 አላውቅም |  |
| 705 | የ PCC ክፍል መገኘት | 1. አዎ  0. የለም |  |
| 707 | በአቅራቢያውወደሚገኝየጤናተቋም (የጤናማእከል፣ሆስፒታል) ለመድረስስንትደቂቃዎች / ኪሎሜትርያህልይወስዳል? | _________ ደቂቃ  ___ ኪሎ ሜትር |  |
| 707 | ወደጤናተቋምበሚሄዱበትጊዜሁሉለጤናማእርግዝናሲባልአገልግሎትአገኙ? | 1. አዎ  0. የለም (ይግለጹ) ______ |  |
| 708 | ቅድመእርዝናእንክብካቤአገልግሎቶችንለማግኘትክፍያከፍለሻል? | 1. አዎ  0. የለም | **የለምከሆነወደ 705ይሂዱ** |
| 709 | 703 መልስአዎከሆነክፍያለእርስዎምንያህልተመጣጣኝነው? | 1. በጣምውድ  2. ሚዛናዊ  3. ርካሽ  4. ነፃ |  |
| 710 | እርጉዝከመሆንዎበፊትየጤናመድንይጠቀሙነበር? | 1. አዎ  0. የለም |  |
| 711 | ለማንኛውምአገልግሎትወደጤናተቋምበሚሄዱበትጊዜሁሉየጤናጥበቃአገልግሎትአቅራቢዎእርጉዝለሆኑሴቶችየሚያስፈልገውንእንክብካቤይነግርዎታል? | 1. አዎ  0. የለም |  |
| 712 | አገልግሎቶችን ለማግኘት የጠፋው ጊዜ | ________ |  |

**ክፍል VIII: ስለቤተሰብዎሃብት**

| ተራቁጥር | ጥያቄ | መልስ | **ወደ… ይሂዱ** |
| --- | --- | --- | --- |
| 801 | ለቤተሰብዎየመጠጥየውሃምንጭከየት ነው? | 1.ከባንባ 2. የተጠበቀ ጉድጓድ  3. ያልተጠበቀ ጉድጓድ 4. የተጠበቀምንጭ  5. ያልተጠበቀ ምንጭ 99. ሌሎች (ይግለጹ)_______ | **ከአንድበላይመልስመስጠትይቻላል** |
| 802 | የቤተሰብዎአባላትምንዓይነትየመጸዳጃቤትይጠቀማሉ? | 1.በነዳጅየተሻሻለየውሃመፀዳጃ 2. ትንሽጉድጓድ ጣሪያ ያለው 3. ትንሽጉድጓድ ጣሪያ የለለው 4. ጫካ  99. ሌላ (ይግለጹ) ___ |  |
| 803 | የእርስዎቤተሰብየሚከተሉ እቃአሉት? | 1.አልጋ 3. መኪና 5. የእጅ ሰአት  2. ቢስክሌት 4. ሞተር ሳይክል 6. ወንበር  7. ሞባይል 8. ጀነሬተር 9. ሬድወ 10. ቴሌቪጅን  11. እሌክትሪክ 12. ሶላር 13. ሶፋ | **ከአንድበላይመልስመስጠትይቻላል** |
| 804 | ወጥቤት የተለየክፍልነው? | 1. አዎ 0. የለም |  |
| 805 | የቤት ወለል ከምንድነው የተሰራ? | 1. ከአፈር 2. እንጨት 3. ሲሚንቶ 4  99. ሌላ (ይግለጹ) ___ | **በማየት የሚሞላ** |
| 806 | የቤት ጣሪ ከምንድነው የተሰራ? | 1.ሣር 2. በቆርቆሮየተሰራ3. ሲሚንቶ  99. ሌላ (ይግለጹ) ___ | **በማየት የሚሞላ** |
| 807 | የቤት ግድግዳ ከምንድነው የተሰራ? | 1. ከጭቃ 2. እንጨት/ጣውላ 3. ሲሚንቶ/ሽክላ  99. ሌላ (ይግለጹ) ___ | **በማየት የሚሞላ** |
| 808 | በአንድክፍልውስጥስንትየቤተሰብአባላትይተኛሉ? | ___________ |  |
| 809 | የቤተሰቡለእርሻስራሊያገለግልየሚችልመሬትአለው? | 1. አዎ 0. የለም |  |
| 810 | ለቡናእርሻስራጥቅምላይሊውልየሚችልየቤተሰቡመሬትአለው? | 1. አዎ 0. የለም |  |
| 811 | የቤተሰቡ የቤትእንስሳትአለው? | 1. አዎ 0. የለም | **አይድለምከሆነወደ 813 ይሂዱ** |
| 812 | ቤተሰቡ ስንት የቤትእንስሳትአለው? | 1. ወተትላሞች_____  2. በሬ___  3. ፈረሶች____  4. በቅሎ______  5. በግ______  6, ዶሮ______  99. ሌላ (ይግለጹ) ___ |  |
| 813 | የቤተሰቡ የባንክሂሳብ አለው? | 1. አዎ 0. የለም |  |
| 814 | 813 መልስአዎከሆነ የቤተሰቡ የባንክሂሳብ ውስጥስንትብርአለዎት? | -----ብር |  |

**እናመሰግናለን!!!**
